# Evaluation of Model-Based Control of Reaction Forces at the Supports of Large-Size Crankshafts

**DOI:** 10.3390/s20092654

**Published:** 2020-05-06

**Authors:** Leszek Chybowski, Krzysztof Nozdrzykowski, Zenon Grządziel, Lech Dorobczyński

**Affiliations:** 1 Faculty of Marine Engineering, Maritime University of Szczecin, ul. Willowa 2-4, 71-650 Szczecin, Poland; k.nozdrzykowski@am.szczecin.pl (K.N.); z.grzadziel@am.szczecin.pl (Z.G.); 2 Faculty of Mechatronics and Electrical Engineering, Maritime University of Szczecin, ul. Willowa 2-4, 71-650 Szczecin, Poland; l.dorobczynski@am.szczecin.pl

**Keywords:** geometry measurements, large crankshafts, marine diesel engines, flexible crankshaft supports, forces at supports, force control, mathematical modeling, changing forces, model-based control

## Abstract

A support control automation system employing force sensors to a large-size crankshaft main journals’ flexible support-system was studied. The current system was intended to evaluate the geometric condition of crankshafts in internal combustion diesel engines. The support reaction forces were changed to minimize the crankshaft elastic deflection as a function of the crank angle. The aim of this research was to verify the hypothesis that the mentioned change can be expressed by a monoharmonic model regardless of a crankshaft structure. The authors’ investigations have confirmed this hypothesis. It was also shown that an algorithmic approach improved the mathematical model mapping with the reaction forces due to faster and more accurate calculations of a phase shift angle. The verification of the model for crankshafts with different structural designs made it possible to assess how well the model fits the coefficients of determination that were calculated with the finite element analysis (FEA). For the crankshafts analyzed, the coefficients of determination R^2^ were greater than 0.9997, while the maximum relative percentage errors δ_max_ were up to 1.0228%. These values can be considered highly satisfactory for the assessment of the conducted study.

## 1. Introduction

The measurement of geometric deviations comprises issues that focus mostly on measuring small-sized components [1,2,3,4]. This limitation is due to the use of small structural components in machinery and mechanisms and the availability of comprehensive instrumentation for measurements. It is also arbitrarily assumed [5,6] that the elastic deflections and deformations of such components due to their own weight are negligible and do not affect the results of measurements. Therefore, this paper does not analyze the practical issues of how an object’s support affects the object’s elastic deformation. In addition, this issue is treated marginally for large components of machinery. This is particularly true for the so-called slender and large-sized components that are of low and variable rigidity with high susceptibility to flexural deformations [7,8]. Important examples of such components are crankshafts of internal combustion diesel engines—the primary units of a main propulsion, ancillary engines and generator sets [9]. More specifically, the crankshafts of piston power machinery used not only in shipbuilding but also in other modes of transport such as railway or automobiles, agriculture, industrial construction and emergency power sources for military facilities and public utilities such as hospitals and offices. These shafts, in addition to their considerable weight and dimensions, have relatively small ratio of cross-section to length. A number of other structural details also make them different from smaller straight shafts used in smaller engines. Another challenge is that these components have different cross-sectional areas along the axis, and the centers of gravity of each section are located at different positions and in different directions relative to the shaft axis.

The known solutions regarding the equipment or systems for the measurement of large-size crankshafts most often involve mounting a shaft on several fixed rigid V-block supports [5,6,10]. For this type of support, it is virtually impossible to obtain reliable measurement results due to initial deflections and geometrical elastic deformations. For these two major reasons, such measurements have elastic deflection errors, whose vectors and magnitude change with the shaft’s rigidity varying as it rotates. Considering the impossibility of eliminating the mentioned deflections in a shaft supported by rigid V-blocks, these deflections are tolerated in this research in order to accommodate the spring action of crank webs [11]. Due to these limitations, the results of spring action measurements are currently the basic indicators for evaluating the correct manufacture of crankshafts.

However, deformation values in large crankshafts can be significant [12,13,14,15] and jeopardize the results of geometric measurements [6,11,16]. Based on our previous research, we considered large crankshafts to be those where the length-to-diameter ratio (L/d) is greater than 12/15, whereas the shape factor α_k_ determining the nature of cross-sectional changes may take on significant values α_k_ > 1 (for a straight shaft with a constant diameter, affected by no sudden changes in cross-section α_k_ = 1) [17]. In such crankshafts deformation, under the influence of their dead weight is checked with usage of deflection (springing) measurements as an indirect measure of bearings reactions. To obtain reliable measurements, these deformations must be analyzed and, where possible, eliminated or minimized, which can be achieved by a combination of suitable object support conditions and appropriate tooling that implements the predetermined support conditions.

Proper conditions for measuring a shaft geometry may be ensured by a measuring system fitted with a so-called “flexible shaft support system”. A design solution for these flexible supports enables the application of variable and pre-set reaction forces. These are predicted with specialized, finite element analysis (FEA) software to ensure zero deflections at crankshaft main journals being supported. The operation of a flexible support system is assisted by a computer application that monitors the variation of the required reaction forces and works together with the automatic control elements. The computer monitoring application records the variation of forces predicted with FEA software. It uses mathematical solvers described previously by the authors in a study where the monoharmonic model was recommended for the item under testing [18].

**Figure 1:**
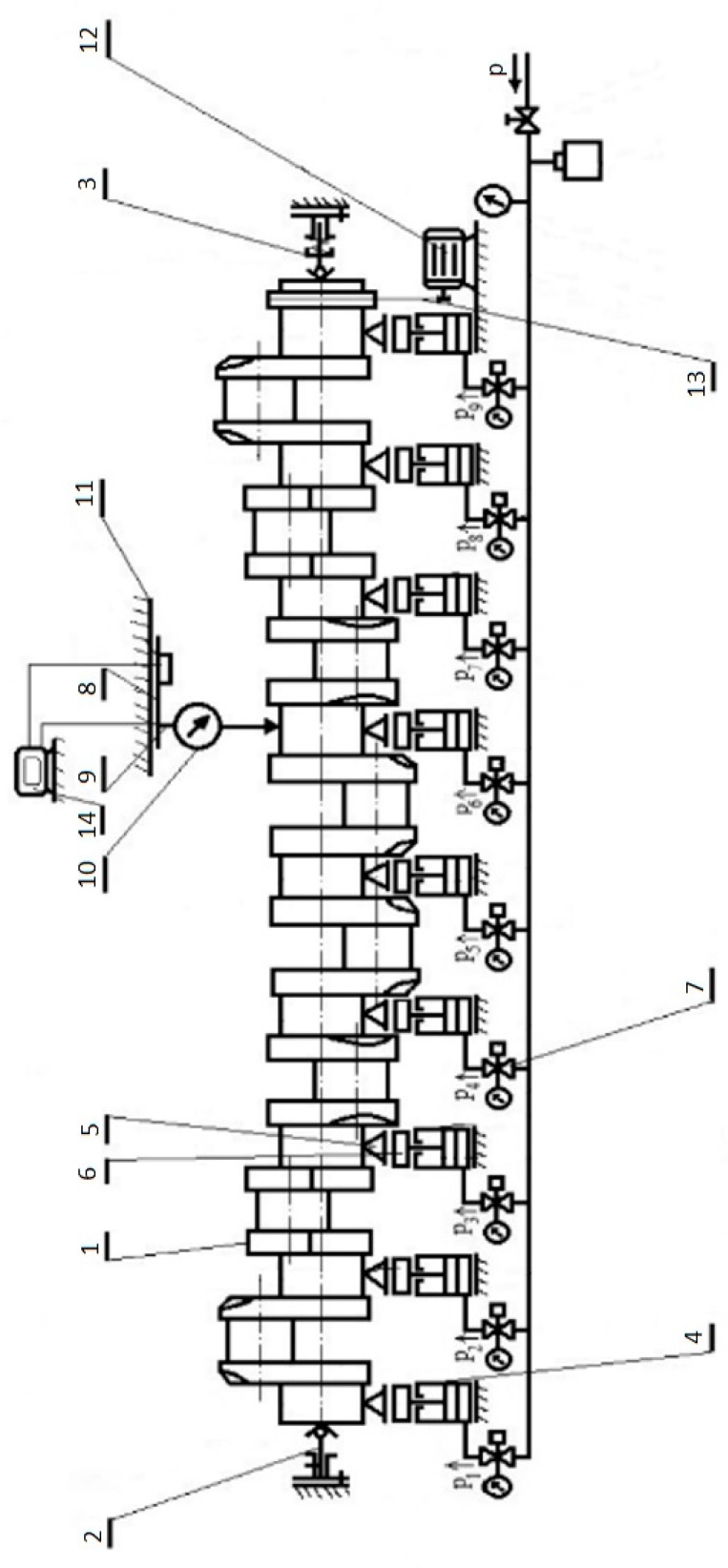
A diagram of the most essential components of the measuring system [6]: (1) crankshaft; (2,3) fixing centers; (4) support with pneumatic actuator; (5) V-block head; (6) force sensor; (7) current-controlled precision valve; (8) trolley; (9) tripod; (10) surface geometry sensor; (11) slide bars; (12) electric motor; (13) belt transmission; (14) PC with software.

According to the experimental results, to accommodate the elastic deflections at the crankshaft, it is necessary to support the main journals during measurements by a set of supports that generate variable reaction forces at the contact of support heads and main journals. To avoid thermal deflections, the temperature of the crankshaft before measurement has to be normalized and must be the same as the ambient temperature. The values of reaction forces should be adjusted to compensate the deflections, both along the shaft axis and at its rotational angle at the supports. A schematic diagram of the main system and essential components of the proposed flexible shaft support system is presented in Figure 1.

**Figure 2:**
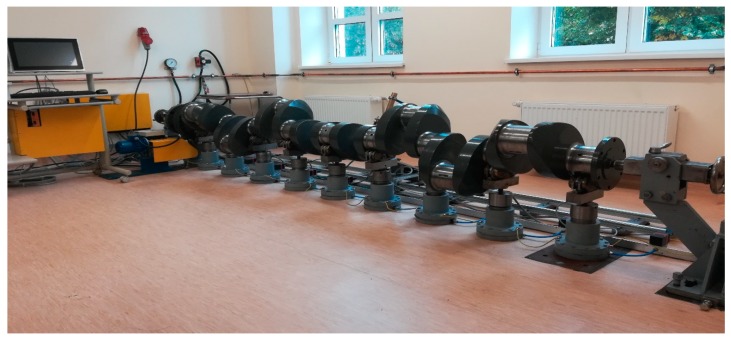
The test rig operating in line with the concept of the measuring system with flexible support of a crankshaft (own source).

The experimental setup is shown in Figure 2. The main system comprises four subsystems: flexible support block, measuring block, turning gear block and the data processing block. The shaft’s flexible support block in the current test rig consists of pneumatic supports (4) fitted with V-block heads (5), force sensors (6) and solenoid valves (7). The measuring block consists of a trolley (8), a tripod and a measuring sensor mounted on the trolley. The trolley moves along the shaft in slide bars (11). The turning gear block generates the crankshaft torque during the measurements. This block consists of an electric motor (12) and a belt transmission (13). The last subsystem is the data processing block, which consists of a computer (14) with the software.

**Figure 3:**
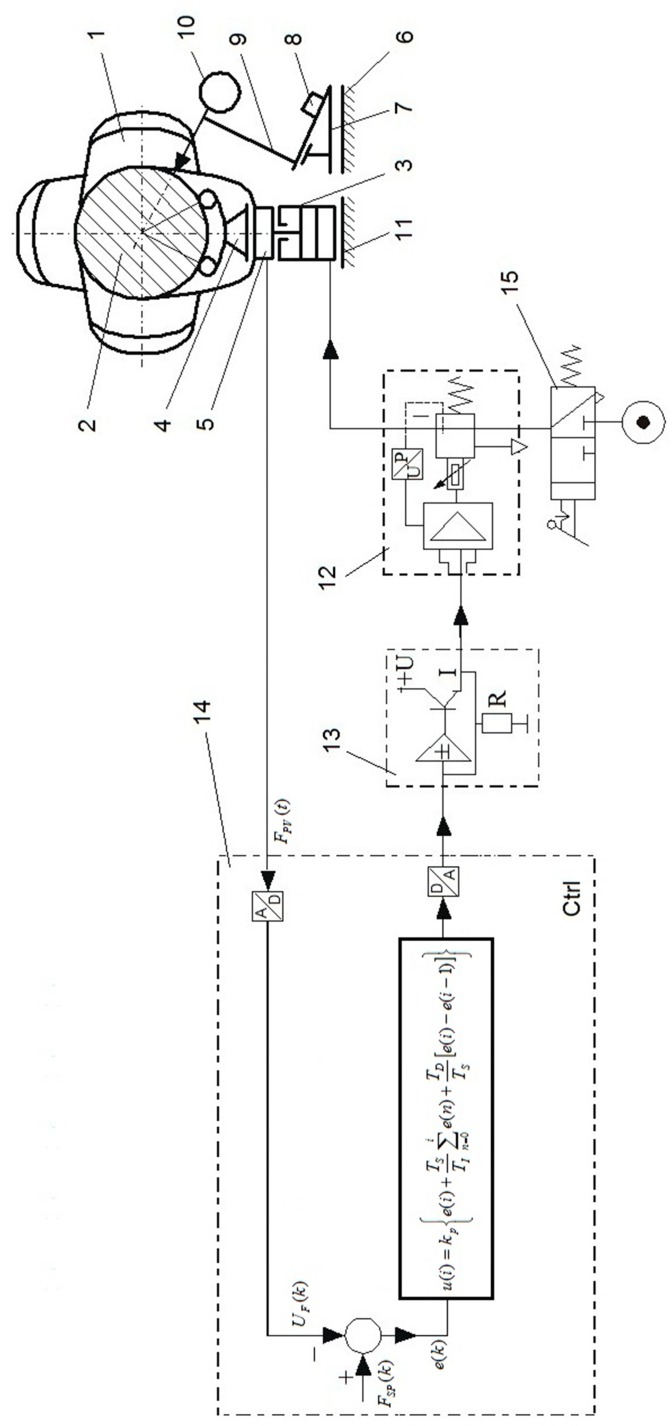
Basic components of the flexible support control unit [11]: (1) crankshaft; (2) shaft’s main journal; (3) pneumatic actuator; (4) rolling, articulated, self-adjusting V-block head; (5) force sensor (force transducer); (6) guides; (7) trolley; (8) laser distance meter for measuring the longitudinal coordinate of the measured cross-section; (9) tripod; (10) surface geometry sensor; (11) base; (12) proportional current-controlled reducing valve (controlled proportional regulating valve); (13) current relay; (14) programmable digital controller (control circuit); (15) feed valve; A, analog signal; Ctrl, controller; D, digital signal; *e*(*k*), an error signal (an input signal of the PID algorithm); *F_PV_*(*t*), signal of realization force; *F_SP_*(*k*), signal of the set force; I, current signal; *kp(i)*, proportional gain; P, pressure; R, resistance; *T_D_*, differentiation time; *T_I_*, integration time; T_S_, sampling time; U, voltage; *U_F_*(*k*), signal corresponding with *F_PV_*(*t*).

The support control algorithm uses a mathematical model that interpolates the values of forces calculated previously with FEA software. The supports are continuously adjusted when the shaft rotates by precision current-controlled valves that operate in feedback with the force sensors measuring the actual force at the contact of support heads and main journals (Figure 3). The whole process is automated using a computer application, developed in-house, to operate the feedback system without any unnecessary delays.

This paper is a continuation of the authors’ studies on the automation of force-sensor-based flexible support of a shaft by main journal actuators. The base control system was presented in [11], and the possible uses of different models of force variation for a selected crankshaft were analyzed in [18]. The purpose of this article is to prove that the most promising monoharmonic model can be used for crankshafts with different designs. Moreover, the use of an algorithmic approach makes it possible to improve the response time of the reaction forces due to a faster and more accurate determination of a phase shift angle, which is one of the elements of the presented model.

Model verification for crankshafts with different designs will allow analyzing the model’s fit and determining the relative percentage errors. In practice, it is assumed that a coefficient of determination (describing how the model fits the given data set) greater than 0.69 renders the fit as substantial [19]. In their earlier papers, the authors assumed that good mapping is provided by the model, for which the coefficient of determination is higher than 0.99 [18].

The authors hypothesize that for a large crankshaft, the change of reaction forces minimizing the elastic deflection of the shaft can be described using a monoharmonic model as a function of the shaft’s angular position, regardless of the number and dimensions of the main and crank journals and the shape, dimensions and angular displacements of cranks.

## 2. Materials and Methods

### 2.1. Monoharmonic Model

The authors’ research to date [18], including the analysis of amplitude spectra of the variation of reaction forces at supports ensuring minimization of elastic deformation of a shaft, has shown that the variation of these forces can be described by the second harmonic. Therefore, the authors suggested using the following monoharmonic model [20]:

(1) $$R\left(\varphi \right)=R_{0}+C_{R2}\sin\left(2\varphi +\varphi_{R2}\right),$$

where *C_R_*_2_ is the amplitude of the second harmonic of the reaction force change function; *φ_R_*_2_ is the phase shift of the second harmonic of the reaction force change function.

Assuming that the reaction forces calculated with the FEA software at the support of a given journal for successive shaft positions are expressed in the form of a vector:

(2) $$\Phi_{FEA}=\left[\varphi_{1},\varphi_{2},\ldots \varphi_{m}\right],$$

(3) $$R_{FEA}=\left[R_{1},R_{2},\ldots R_{m}\right],$$

Then, the individual constants of function (1) will be [20]:

(4) $$R_{0}=\frac{\sum_{i=1}^{m}R_{i}}{m},$$

(5) $$C_{R2}=\frac{\underset{i-1,2,\ldots m}{\max}(R_{1},R_{2},\ldots R_{m})-\underset{i-1,2,\ldots m}{\min}\left(R_{1},R_{2},\ldots R_{m}\right)}{2},$$

(6) $$\varphi_{R2}=\arcsin\left(\frac{R_{i}-R_{0}}{C_{R2}}\right)-2\varphi_{i},$$

(7) $$\left(\frac{R_{i}-R_{0}}{C_{R2}}\right)\in -1,1,$$

where *ϕ_i_* is the angle for which the phase shift is determined.

When determining the phase shift, it is important that the variation of the model coincides with the variation of the curve evaluated by the FEA software. For example, both characteristics should be increasing for the shaft position corresponding to the angle *φ_i_*. For the values calculated for the shaft of Buckau Wolf R8VD-136 engine [18], *φ_i_* = 0°CA for even journals and *φ_i_* = 45°CA for odd journals. In general, the analytical determination of the phase shift angle uses the methods given by Mateusz Kowalski [21], modified by the authors.

### 2.2. The Analytical Method for Determining the Phase Shift

The fit of the model to the FEA calculation results can be described using the quality function of the unfitness *χ*^2^(*ϕ*) [22], which should be as small as possible (the higher the value of *χ*^2^(*ϕ*), the worse the fit). This function is given by the formula:

(8) $$\chi^{2}\left(\varphi \right)=\sum_{i=1}^{m}\frac{{\left\{R_{i}-\left[R_{0}+C_{R2}\sin(2\varphi_{i}-\varphi_{R2}\right]\right\}}^{2}}{\sigma_{i}^{2}},$$

The inaccuracy of calculated individual reaction forces is considered constant, hence *σ = σ*_1_ = *σ*_2_ *= … = σ_i_ = … σ_m_*. Thus, relationship (8) takes the form:

(9) $$\chi^{2}\left(\varphi \right)=\frac{1}{\sigma }\sum_{i=1}^{m}{\left\{R_{i}-\left[R_{0}+C_{R2}\sin(2\varphi_{i}-\varphi_{R2}\right]\right\}}^{2},$$

The fit of FEA data to a model reaches an extreme for [*χ*^2^(*ϕ*)]’ = 0, i.e.,

(10) $$\sum_{i=1}^{m}\left[R_{i}\cos\left(2\varphi_{i}+\varphi_{R2}\right)-\sin\left(2\varphi_{i}+\varphi_{R2}\right)\cos\left(2\varphi_{i}+\varphi_{R2}\right)\right]=0,$$

Having applied the sum formula for cosine and the sum formula for sine [20]:

(11) $$\cos\left(2\varphi_{i}+\varphi_{R2}\right)=\cos\left(2\varphi_{i}\right)\cos\left(\varphi_{R2}\right)-\sin\left(2\varphi_{i}\right)\sin\left(\varphi_{R2}\right),$$

(12) $$\begin{array}{l} \sin\left(2\varphi_{i}+\varphi_{R2}\right)\cos\left(2\varphi_{i}+\varphi_{R2}\right)=\\ \;\;\;\;\;\;=\frac{1}{2}\cos^{2}\left(\varphi_{R2}\right)\sin\left(4\varphi_{i}\right)+\sin\left(\varphi_{R2}\right)\cos\left(\varphi_{R2}\right)\cos\left(4\varphi_{i}\right)\\ \;\;\;\;\;\;-\frac{1}{2}sin^{2}\left(\varphi_{R2}\right)sin\left(4\varphi_{i}\right), \end{array}$$

Relationship (10) takes the form:

(13) $$\begin{array}{c} \sum_{i=1}^{m}\{R_{i}\cos\left(2\varphi_{i}\right)\cos\left(\varphi_{R2}\right)-R_{i}\sin\left(2\varphi_{i}\right)\sin\left(\varphi_{R2}\right)-[\frac{1}{2}\cos^{2}\left(\varphi_{R2}\right)\sin\left(4\varphi_{i}\right)+\\ \sin\left(\varphi_{R2}\right)\cos\left(\varphi_{R2}\right)\cos\left(4\varphi_{i}\right)-\frac{1}{2}sin^{2}\left(\varphi_{R2}\right)sin\left(4\varphi_{i}\right)]\}=0, \end{array}$$

Relationship (13) can be expressed as the sum of sums [21]:

(14) $$\begin{array}{c} \cos\left(\varphi_{R2}\right)\sum_{i=1}^{m}R_{i}\cos(2\varphi_{i})-\sin\left(\varphi_{R2}\right)\sum_{i=1}^{m}R_{i}\sin(2\varphi_{i})-\frac{1}{2}\cos^{2}\left(\varphi_{R2}\right)\sum_{i=1}^{m}\sin\left(4\varphi_{i}\right)-\\ \sin\left(\varphi_{R2}\right)\cos\left(\varphi_{R2}\right)\sum_{i=1}^{m}\cos\left(4\varphi_{i}\right)+\frac{1}{2}\sin^{2}\left(\varphi_{R2}\right)\sum_{i=1}^{m}\sin\left(4\varphi_{i}\right)=0, \end{array}$$

After substituting:

(15) $$A=\sum_{i=1}^{m}R_{i}\cos\left(2\varphi_{i}\right),$$

(16) $$B=\sum_{i=1}^{m}R_{i}\sin\left(2\varphi_{i}\right),$$

(17) $$C=\sum_{i=1}^{m}\sin\left(4\varphi_{i}\right),$$

(18) $$D=\sum_{i=1}^{m}\cos\left(4\varphi_{i}\right),$$

relationship (14) takes the form [21]:

(19) $$A\cos\left(\varphi_{R2}\right)-B\sin\left(\varphi_{R2}\right)-\frac{1}{2}C\cos^{2}\left(\varphi_{R2}\right)-D\sin\left(\varphi_{R2}\right)\cos\left(\varphi_{R2}\right)+\frac{1}{2}\sin^{2}\left(\varphi_{R2}\right)=0,$$

Using the identity $\sin^{2}\left(\varphi_{R2}\right)+\cos^{2}\left(\varphi_{R2}\right)=1$ and raising both sides of Equation (19) to the second power, we get [21]:

(20) $$\begin{array}{c} \sin^{4}\left(\varphi_{R2}\right)\left(C^{2}+D^{2}\right)+\sin^{3}\left(\varphi_{R2}\right)\left(-2BC-2AD\right)+\sin^{2}\left(\varphi_{R2}\right)\left(B^{2}-C^{2}+A^{2}-D^{2}\right)+\\ \sin\left(\varphi_{R2}\right)\left(BC+2AD\right)+\left(\frac{C^{2}}{4}-A^{2}\right)=0, \end{array}$$

The next stage of calculation is to solve Equation (20). This can be done by the Ferro method [23] or numerically, using the roots function in MATLAB 2019b (MathWorks, Natick, MA, USA) or Octave 5.1.0 (John W. Eaton et al., GNU General Public License—GPL) [24]. Then, values $w=\sin\left(\varphi_{R2}\right)$ are determined, with which, for real roots, the values of potential candidates must be determined for the correct phase shift angle:

(21) $$\varphi_{R2}=\arcsin\left(w\right),$$

Finally, it is necessary to verify which extremes are minimums of the unfitness function, which requires calculating expression (8) and incorporating the value for which the unfitness function is the smallest into the model.

### 2.3. The Recursive Method for Determining the Phase Shift

**Figure 4:**
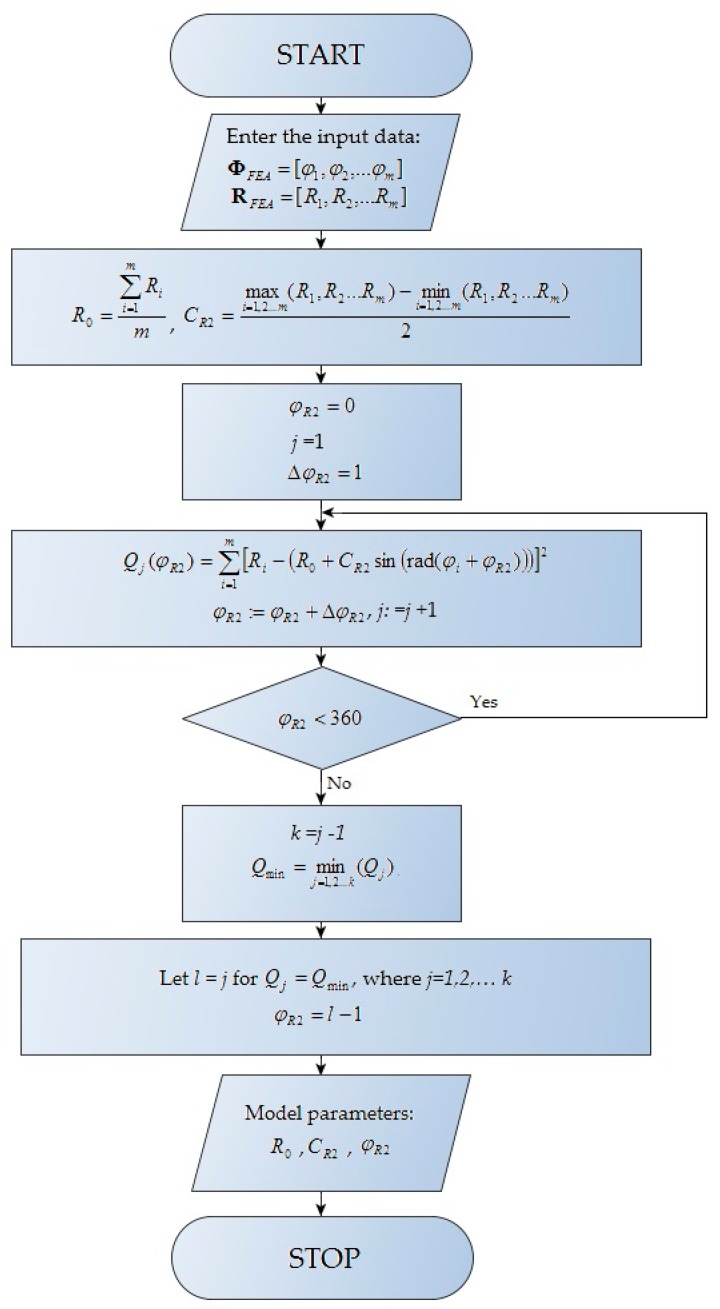
Recursive algorithm for determination of model parameters.

An alternative to the analytical solution is determining the phase shift angle using a recursive algorithm. This solution simplifies the search for the constants of a model and decreases the evaluation time. The authors used their experience to make an educated guess, assuming that it would be sufficient to determine the crank angle to an accuracy of 1° (CA). The proposed calculation algorithm for a single main journal is presented in Figure 4.

The algorithm was implemented as code for MATLAB 2019b (MathWorks, Natick, MA, USA) and is presented in Appendix A.

To evaluate the usability of the proposed monoharmonic model and the algorithm presented in the paper, the model fit results and FEA calculations were analyzed and compared. The FEA calculations were done using the Midas NFX 2019 R1 (MSC Software Corporation, Newport Beach, CA, USA).

**Figure A1:**
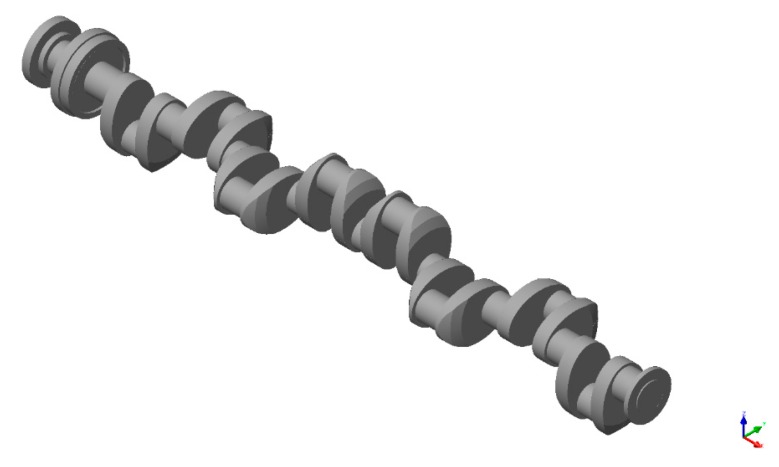
A 3D model of the crankshaft S-9285-3600-10-8-149-144-O-01.

**Figure A2:**
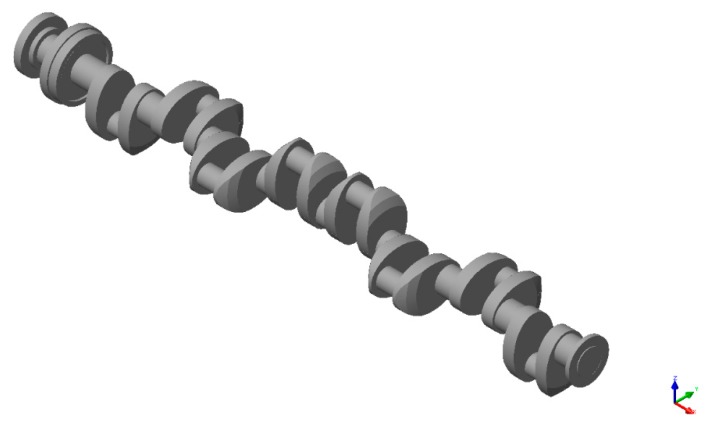
A 3D model of the crankshaft S-8658-3600-10-8-149-114-O-02.

**Figure A3:**
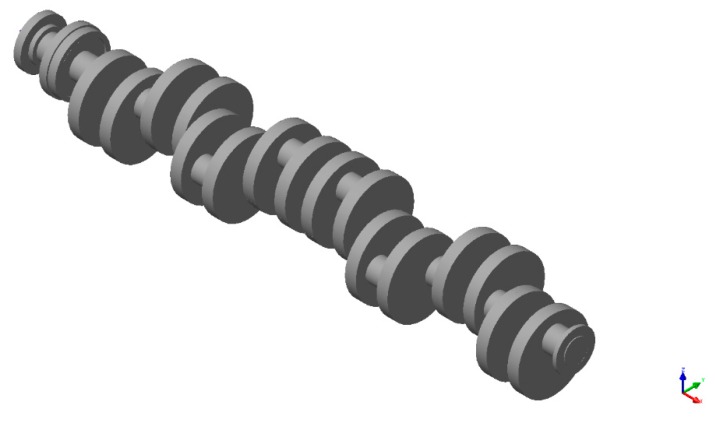
A 3D model of the crankshaft S-16942-3600-10-8-149-144-C-03.

**Figure A4:**
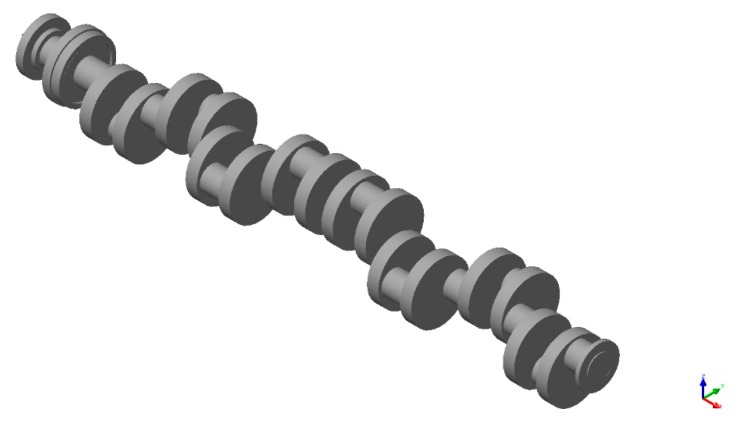
A 3D model of the crankshaft S-12075-3600-10-8-149-144-C-04.

**Figure A5:**
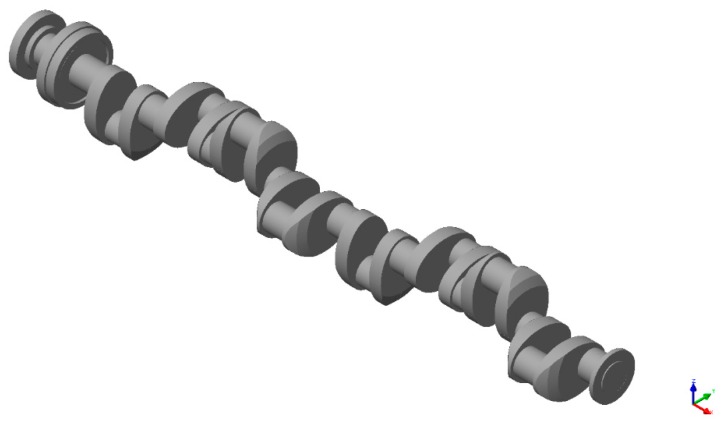
A 3D model of the crankshaft S-9283-3600-10-8-149-144-O-05.

**Figure A6:**
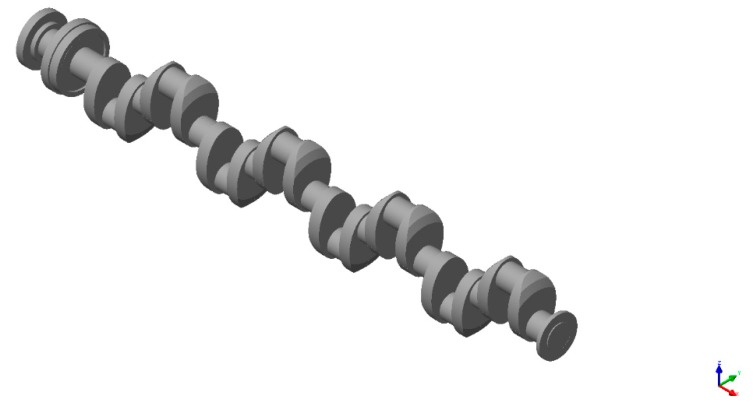
A 3D model of the crankshaft S-9283-3600-10-8-149-144-O-06.

**Figure A7:**
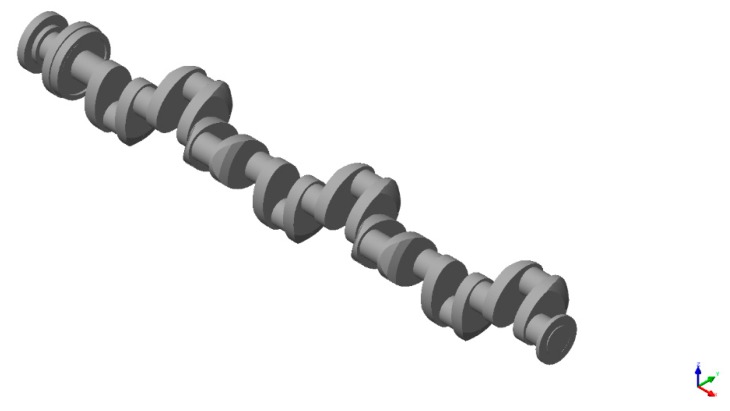
A 3D model of the crankshaft S-9283-3600-10-8-149-144-O-07.

**Figure A8:**
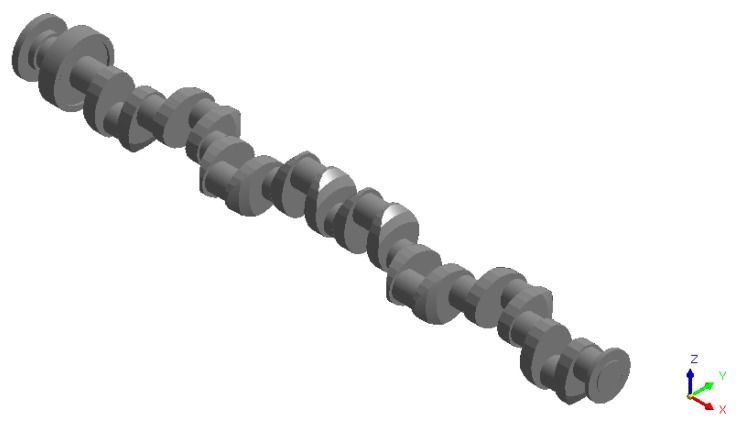
A 3D model of the crankshaft S-8479-3600-10-8-149-144-O-08.

**Figure A9:**
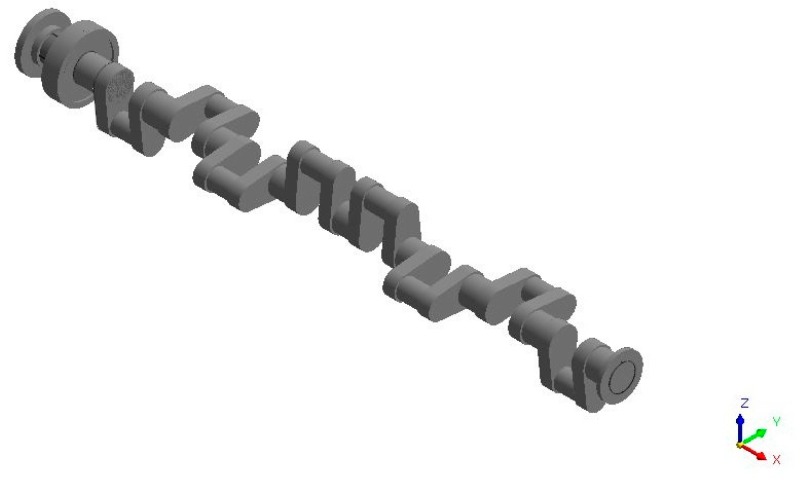
A 3D model of the crankshaft S-7051-3600-10-8-149-114-Z-09.

**Figure A10:**
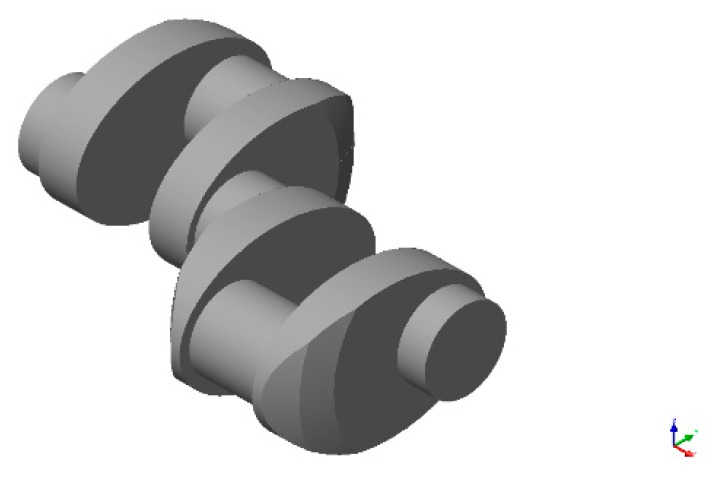
A 3D model of the crankshaft S-1977-0740-3-2-149-144-O-10.

**Figure A11:**
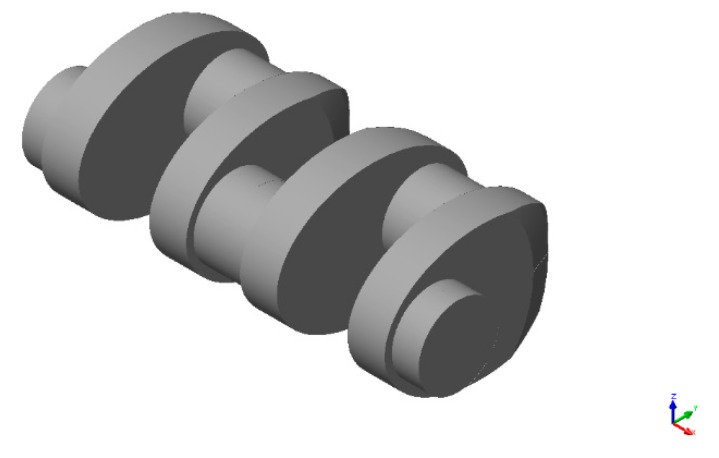
A 3D model of the crankshaft S-1977-0740-3-2-149-144-O-11.

**Figure A12:**
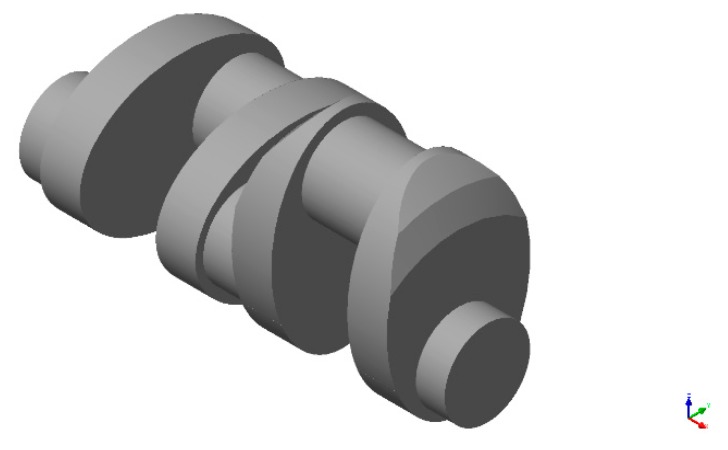
A 3D model of the crankshaft S-1977-0740-3-2-149-144-O-12.

At main journal supports of shafts, the active forces are analyzed, and reaction forces are generated to minimize the elastic deflection of the shaft for individual angular positions of the shaft. These forces are compiled in Appendix B, in Table A1, Table A2, Table A3, Table A4, Table A5, Table A6, Table A7, Table A8, Table A9, Table A10, Table A11 and Table A12. The 3D models of analyzed crankshafts are presented in Appendix C, in Figure A1, Figure A2, Figure A3, Figure A4, Figure A5, Figure A6, Figure A7, Figure A8, Figure A9, Figure A10, Figure A11 and Figure A12. The graphical presentation of FEA results for the selected crankshaft designs is provided in the paper. The analysis assumes that the initial position of 0°CA corresponds to the top dead center (TDC) of the first journal on the flywheel side (the assumption was made according to the specification of the Buckau Wolf R8VD-136 engine). The direction of shaft rotation was assumed to be clockwise when viewed from the free end of the shaft.

The authors proposed a monoharmonic model with coefficients calculated in a recursive algorithm, which was implemented in the form of a code in MATLAB 2019b (MathWorks, Natick, MA, USA).

In order to consider different design options and assess their impact on the extent of applicability of the monoharmonic model proposed, the authors analyzed eight crankshafts with 10 main journals and three crankshafts with three main journals. To ensure the traceability, shafts were labeled in the following manner to provide information on their selected dimensions: S-MMMMM-XXXX-YY-ZZ-AAA-BBB-D-EE. Here, individual terms are defined as follows: S, shaft; MMMMM, shaft weight in newton (N); XXXX, shaft length in millimeters (mm); YY, number of main journals; ZZ, number of crank journals; AAA, main journal diameter in millimeters (mm); BBB, crank journal diameter in millimeters (mm); D, shape of crank webs (O—oval, C—circular, Z—figure formed by two circles connected by tangents); EE, specific version including crank dimensions, their relative angular offsets and the total shaft weight.

The coefficients of determination *R*^2^ and maximum relative percentage error *δ*_max_ were determined for each main journal to assess the accuracy of the monoharmonic model’s fit for the reaction forces. Finally, the results were obtained, and conclusions were drawn for the suitability of the proposed models.

## 3. Results and Discussion

### 3.1. Crankshaft S-9285-3600-10-8-149-144-O-01

The first crankshaft analyzed was the shaft of the Buckau Wolf R8DV-136 (manufacturer: VEB SKL—Magdeburg, number of cylinders: 8, nominal effective power: 220 kW, nominal speed: 360 rpm, nominal specific fuel oil consumption: 238 g/kWh, cylinder bore: 240 mm, piston stroke: 360 mm). This was done to validate the accuracy of the model. The basic parameters of the shaft are as follows: weight of 9285 N, length of 3600 mm, ten main journals 149 mm in diameter, eight crank journals 144 mm in diameter, journal length of 100 mm, oval crank webs measuring 252 mm × 358 mm. Reaction force monoharmonic model coefficients that minimize the elastic deflection are listed in Table 1.

The quality of the model’s fit to the FEA values and relative percentage errors of the reaction forces are presented in Table 2.

The smallest coefficient of determination, equal to 0.9999, corresponds to the model of reaction forces at the support of the main journal No. 3, while the largest relative percentage error of the reaction forces, equal to 0.7788%, corresponds to the model of reaction forces at the support of the main journal No. 5.

### 3.2. Crankshaft S-8658-9285-3600-10-8-149-114-O-02

Subsequently, the shaft that was redesigned by modifying the journal diameter was analyzed. The basic parameters of the shaft are as follows: weight of 8658 N, length of 3600 mm, ten main journals 149 mm in diameter, eight crank journals 114 mm in diameter, journal length of 100 mm, oval crank webs measuring 252 mm × 358 mm. Reaction force monoharmonic model coefficients that minimize the elastic deflection are listed in Table 3.

The quality of the model’s fit to the FEA values and relative percentage errors of reaction forces are presented in Table 4.

The smallest coefficient of determination, equal to 0.9999, corresponds to the model of reaction forces at the support of the main journal No. 1, while the largest relative percentage error of the reaction forces, equal to 1.0481%, corresponds to the model of reaction forces at the support of the main journal No. 5.

### 3.3. Crankshaft S-16942-3600-10-8-149-144-C-03

Next, the shaft that was redesigned by modifying the crank shape was analyzed. The basic parameters of the shaft are as follows: weight of 16,942 N, length of 3600 mm, ten main journals with a diameter of 149 mm, eight crank journals with a diameter of 144 mm, journal length of 100 mm, circular crank webs 450 mm in diameter. Reaction force monoharmonic model coefficients that minimize the elastic deflection are listed in Table 5.

The quality of the model’s fit to the FEA values and relative percentage errors of reaction forces are presented in Table 6.

The smallest coefficient of determination, equal to 0.9999, corresponds to the model of reaction forces at the support of the main journal No. 4, while the largest relative percentage error of the reaction forces, equal to 0.6581%, corresponds to the model of reaction forces at the support of the main journal No. 5.

### 3.4. Crankshaft S-12075-3600-10-8-149-144-C-04

Subsequently, the analysis was done for a shaft with a design as in the case of the shaft S-12075-3600-10-8-149-144-C-03, but with a reduced diameter of crank webs. The basic parameters of the shaft are as follows: weight of 12,075 N, length of 3600 mm, ten main journals with a diameter of 149 mm, eight crank journals with a diameter of 144 mm, journal length of 100 mm, circular crank webs 358 mm in diameter. Reaction force monoharmonic model coefficients that minimize the elastic deflection are listed in Table 7.

The quality of the model’s fit to the FEA values and relative percentage errors of reaction forces are presented in Table 8.

The smallest coefficient of determination, equal to 0.9999, corresponds to models of reaction forces at the support of the main journals Nos. 1, 4, 7 and 10, while the largest relative percentage error of the reaction forces, equal to 0.5482%, corresponds to the model of reaction forces at the support of the main journal No. 5.

### 3.5. Crankshaft S-9283-3600-10-8-149-144-O-05

The redesigned shaft with relative angles between cranks modified by 90° was then analyzed. The basic parameters of the shaft are as follows: weight of 9283 N, length of 3600 mm, ten main journals of 149 mm diameter, eight crank journals 144 mm in diameter, journal length of 100 mm, oval crank webs measuring 252 mm × 358 mm, offset by the angle of 90° relative to each other. Reaction force monoharmonic model coefficients that minimize the elastic deflection are listed in Table 9.

The quality of the model’s fit to the FEA values and relative percentage errors of reaction forces are presented in Table 10.

The smallest coefficient of determination, equal to 0.9999, corresponds to models of reaction forces at the support of the main journals Nos. 3, 6 and 8, while the largest relative percentage error of the reaction forces, equal to 0.4026%, corresponds to the model of reaction forces at the support of the main journal No. 5.

### 3.6. Crankshaft S-9283-3600-10-8-149-144-O-06

Subsequently, the analysis was carried out for a shaft redesigned by modifying the relative angles between cranks with a 180° offset in succession. The basic parameters of the shaft are as follows: weight of 9283 N, length of 3600 mm, ten main journals 149 mm in diameter, eight crank journals 144 mm in diameter, journal length of 100 mm, oval crank webs measuring 252 mm × 358 mm, offset by the angle of 180° relative to each other. Reaction force monoharmonic model coefficients that minimize the elastic deflection are listed in Table 11.

The quality of the model’s fit to the FEA values and relative percentage errors of reaction forces are presented in Table 12.

The smallest coefficient of determination, equal to 0.9999, corresponds to the model of reaction forces at the support of the main journal No. 2, while the largest relative percentage error of the reaction forces, equal to 0.0887%, corresponds to the model of reaction forces at the support of the main journal No. 2.

### 3.7. Crankshaft S-9283-3600-10-8-149-144-O-07

Subsequently, the analysis was carried out for a shaft redesigned by modifying the relative angles between cranks with a 120° offset in succession. The basic parameters of the shaft are as follows: weight of 9283 N, length of 3600 mm, ten main journals 149 mm in diameter, eight crank journals 144 mm in diameter, journal length of 100 mm, oval crank webs measuring 252 mm × 358 mm, offset by the angle of 120° relative to each other. Reaction force monoharmonic model coefficients that minimize the elastic deflection are listed in Table 13.

The quality of the model’s fit to the FEA values and relative percentage errors of reaction forces are presented in Table 14.

The smallest coefficient of determination, equal to 0.9999, corresponds to models of reaction forces at the support of the main journals Nos. 2, 5 and 9, while the largest relative percentage error of the reaction forces, equal to 0.3983%, corresponds to the model of reaction forces at the support of the main journal No. 2.

### 3.8. Crankshaft S-8479-3600-10-8-149-144-O-08

Next, an analysis was carried out for the shaft with a design modified by increasing the so-called drive ratio of main and crank journals, which was done by reducing the distance between the axis of the main and crank journals. The basic parameters of the shaft are as follows: weight of 8479 N, length of 3600 mm, ten main journals 149 mm in diameter, eight crank journals 144 mm in diameter, journal length of 100 mm, oval crank webs measuring 252 mm × 358 mm, reduced distance between main journal axis and crank journal axis. Reaction force monoharmonic model coefficients that minimize the elastic deflection are listed in Table 15.

The quality of the model’s fit to the FEA values and relative percentage errors of reaction forces are presented in Table 16.

The smallest coefficient of determination, equal to 0.9999, corresponds to models of reaction forces at the support of the main journals Nos. 1, 6, 7 and 10, while the largest relative percentage error of the reaction forces, equal to 0.4840%, corresponds to the model of reaction forces at the support of the main journal No. 5.

### 3.9. Crankshaft S-7051-3600-10-8-149-144-Z-09

Next, the shaft was redesigned in relation to the shaft S-8658-3600-10-8-149-144-C-03 to form a complex shape consisting of two circles connected by two tangents. The basic parameters of the shaft are as follows: weight of 7051 N, length of 3600 mm, ten main journals 149 mm in diameter, eight crank journals 144 mm in diameter, journal length of 100 mm, crank webs with a complex shape of maximum dimensions 168 mm × 331 mm. Reaction force monoharmonic model coefficients that minimize the elastic deflection are listed in Table 17.

The quality of the model’s fit to the FEA values and relative percentage errors of reaction forces are presented in Table 18.

The smallest coefficient of determination, equal to 0.9999, corresponds to the model of reaction forces at the support of the main journal No. 2, while the largest relative percentage error of the reaction forces, equal to 1.0228%, corresponds to the model of reaction forces at the support of the main journal No. 5.

### 3.10. Crankshaft S-1977-0740-3-2-149-144-O-10

Subsequently, the analysis was carried out on a shaft with double crank and with crank webs that were offset by 180°. The basic parameters of the shaft are as follows: weight of 1977 N, length of 740 mm, three main journals with a diameter of 149 mm, outermost journal length of 50 mm, other journals’ length of 100 mm, two crank journals with a diameter of 144 mm, oval crank webs measuring 252 mm × 358 mm located on the opposite sides of one plane. Reaction force monoharmonic model coefficients that minimize the elastic deflection are listed in Table 19.

The quality of the model’s fit to the FEA values and relative percentage errors of reaction forces are presented in Table 20.

The coefficients of determination equal 1.0000 for all journals, being accurate to 4 decimal places, while the largest relative percentage error of the reaction forces equals 0.0159%, which corresponds to the model of reaction forces at the support of the main journal No. 1.

### 3.11. Crankshaft S-1977-0740-3-2-149-144-O-11

Next, the analysis was carried out on a shaft with a double crank and crank journal positions allocated at the same axis. The basic parameters of the shaft are as follows: weight of 1977 N, length of 740 mm, three main journals with a diameter of 149 mm, outermost journal length of 50 mm, other journals’ length of 100 mm, two crank journals with a diameter of 144 mm, oval crank webs measuring 252 mm × 358 mm located in one plane, on the same side. Reaction force monoharmonic model coefficients that minimize the elastic deflection are listed in Table 21.

The quality of the model’s fit to the FEA values and relative percentage errors of reaction forces are presented in Table 22.

The coefficients of determination equal 0.9997 for all journals, while the largest relative percentage error of the reaction forces equals 0.2077%, which corresponds to models of reaction forces at the support of main journals Nos. 1 and 3.

### 3.12. Crankshaft S-1977-0740-3-2-149-144-O-12

The last analysis was carried out on a shaft with a double crank and crank webs offset by 90°. The basic parameters of the shaft are as follows: weight of 1977 N, length of 740 mm, three main journals with a diameter of 149 mm, outermost journal length of 50 mm, other journals’ length of 100 mm, two crank journals with a diameter of 144 mm, oval crank webs sized 252 mm × 358 mm, mutually perpendicular. Reaction force monoharmonic model coefficients that minimize the elastic deflection are listed in Table 23.

The quality of the model’s fit to the FEA values and relative percentage errors of reaction forces are presented in Table 24.

The coefficients of determination equal 1.0000 for all journals, being accurate to 4 decimal places, similar to the previous case, while the largest relative percentage error of the reaction forces equals 0.3143%, which corresponds to the model of reaction forces at the support of the main journal No. 3.

### 3.13. Comparative Analysis

The results obtained for all 12 crankshafts under analysis are compared in Table 25; for each model, the maximum relative error *δ_max_* and the minimum coefficient of determination R^2^_min_ are listed. The calculated results for the monoharmonic model based on the second harmonic for all 12 crankshafts with different designs are found to be in complete agreement the FEA data. For all crankshafts analyzed, the coefficients of determination *R*^2^ are greater than 0.9997, and this value is valid for all main journals of the crankshaft S-1977-0740-3-2-149-144-O-11. The maximum relative percentage error does not exceed 1.0228%, which is valid for the main journal No. 5 of the crankshaft S-7051-3600-10-8-149-144-Z-09.

The analysis has shown that the monoharmonic model maps the reaction forces at supports of main journals, regardless of how the shaft is designed. In particular, the designs were changed by modifying the following features:

- the number of crankshaft’s main journals,
- the number of crankshaft’s crank journals,
- the length of main journals,
- the diameter of main journals,
- the diameter of crank journals,
- dimensions of the crank webs,
- the shape of crank webs,
- the relative angular offset between subsequent crank webs.

As the phase shift angle was determined using model algorithms, there was a significant improvement in the quality of the model’s fit to the FEA data. This is true for an angular increment of 15°. With the algorithm, it is possible to increase the number of interpolation nodes, as shown in this article for an angular step of 1°. The authors’ previous studies [18] have shown calculations for every 15° and the phase shift given by the formula (6), the shaft S-9283-3600-10-8-149-144-O-01 was modeled with the fit resulting in the coefficient of determination R^2^_min_ = 0.9959 and the maximum relative percentage error *δ_max_* = 1.52899. Thus, according to the data presented in Table 25, the refinement of interpolation to 360 nodes per 1 revolution allowed reducing the error by less than 51%.

## 4. Model Validation

The operational tests for verification of the proposed system were conducted using a test rig built in the Szczecin AM, equipped with the flexible shaft support system presented in the Section 1.

The testing included measurements of the geometry deviations in main journals of the crankshaft of a Buckau Wolf R8DV-136 engine (crankshaft designated S-9285-3600-10-8-149-144-O-01). Measurements were carried out in which the crankshaft was supported by a set of rigid V-block supports and also with the shaft being supported by a set of flexible supports. The outer faces of the shaft were fixed at the centers. For this variant, at the shaft support points, we calculated the reaction forces beforehand to ensure zero deflection at the main journals, in 15 angular degree increments. Then, these values were replaced with the model given in Section 2.3, and the measurement results were compared with the assumed reference results. The reference measurements were performed using an operative measuring system with a MUK 25-600 measuring head equipped with SAJD software that was previously tested under industrial conditions.

The reference measurement system was designed to measure the roundness profiles of cylindrical surfaces using a reference-based method. The MUK 25-600 head was seated directly on the surface of the tested journal and assessed the shape profile independent of the measured item’s support conditions. This system was selected for reference measurements because we could compare the measurements using similar mathematical tools, including harmonic analysis of profiles. The measurement procedure was based on polar coordinates. In individual cross-sections, consecutive changes in the radius *r_ji_* of a specific angle of shaft rotation *φ_ji_* were measured.

The roundness profiles, reference *r*_1_(*φ*) and corresponding tested one *r*_2_(*φ*), were pre-filtered for harmonics in the range of *n* = 2–15 and then comparatively evaluated using a standardized intercorrelation function given by:

(22) $$\rho \left(\gamma_{\phi }\right)=\frac{2\int_{0}^{2\pi }r_{1}\left(\phi \right)r_{2}\left(\phi +\gamma_{\phi }\right)d\phi }{\int_{0}^{2\pi }r_{1}{\left(\phi \right)}^{2}d\phi +\int_{0}^{2\pi }r_{2}{\left(\phi \right)}^{2}d\phi }$$

where: *r_1_(φ)* is the roundness profile measured with the reference method, *r_2_(φ)* is the roundness profile measured by the evaluated method and $\gamma_{\varphi }$ is the phase shift between the graphs being compared.

**Figure 5:**
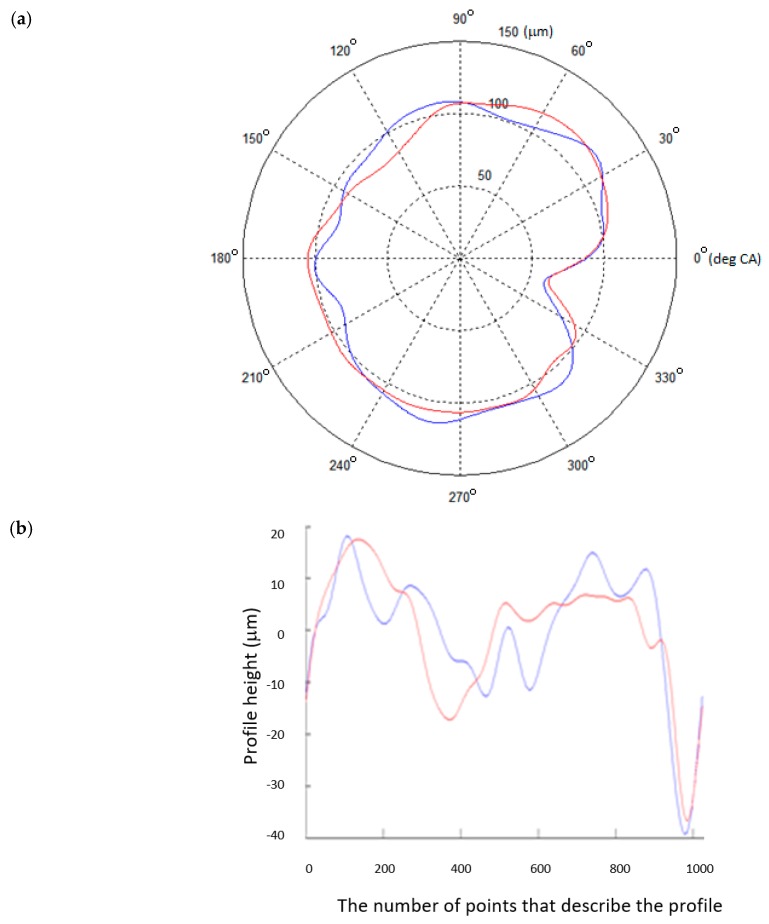
The measured (blue) and reference (red) profiles of journal No. 4, obtained for a shaft supported by a set of rigid V-blocks: (**a**) in the polar coordinate system; (**b**) in the Cartesian coordinate system.

For a shaft supported by a set of rigid V-blocks, the compared profiles showed moderate overlap. The coefficient of intercorrelation between the tested journals had values from 0.7665 to 0.8132. This is shown qualitatively in Figure 5 using superimposed measured and reference roundness profiles mapped in polar and Cartesian coordinates for the selected main journal (No. 4). The intercorrelation determined for this journal was *ρ*(*γ_φ_*) = 0.7987.

**Figure 6:**
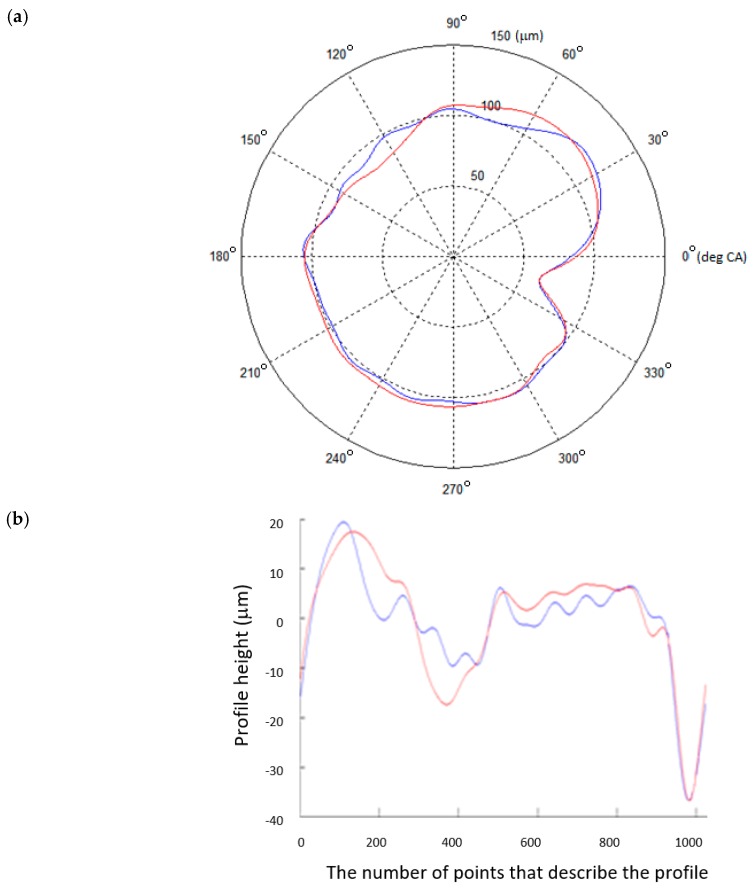
The measured (blue) and reference (red) profiles of journal No. 4 obtained for the controlled-reaction-force shaft support: (**a**) in polar coordinates; (**b**) in Cartesian coordinates.

The measurements on a flexible shaft support, assisted by the algorithm described in Section 2.3, were compared with the reference measurements (Figure 6). As in the preceding case, the comparison is made in both polar and Cartesian coordinates.

When we applied the proposed algorithm to a shaft support with controlled reaction forces, the results showed a high correlation between the compared profiles. The intercorrelation coefficient between the tested journal profiles ranged from 0.9113 to 0.9399. The intercorrelation coefficient of main journal No. 4 was determined to be *ρ*(*γ_φ_*) = 0.9266.

The experimental studies have confirmed the suitability of the presented models. This is a major step in the development of a geometry measurement system with flexible shaft support for large-size crankshafts.

## 5. Conclusions

The monoharmonic model provides results which are in agreement with the calculated data. These values can be used in the algorithmic control of reaction forces at the supports of a crankshaft geometry measuring system. The model has higher degree of accuracy and simplicity compared to the other potential options such as the basic polyharmonic model and spline-based polyharmonic model. The current model is not dependent on spectral analysis of the input data to describe the individual harmonics for the function of reaction force variation calculated with the FEA software.

It should be noted that for all modifications in the design of shafts analyzed, the common feature is the symmetry of the crank webs relative to the plane that contains the longitudinal axis of the main journal and crank journal, which are located in the immediate vicinity of the crank in question. Therefore, it can be said that it is possible to maintain the regularity for virtually all large-size crankshafts used in modern internal combustion high-power engines.

The proposed models make it possible to automate the control of flexible supports of crankshaft’s main journals, thereby increasing the accuracy of the measurement process by minimizing the elastic deflection of the measured shaft.

## 6. Patents

1. Chybowski L., Kazienko D., *Universal resistance system supporting the calibration of resistance sensors in energetic machines monitoring systems, preferably internal combustion engines*. Polish Patent Office, P.429574.
2. Chybowski L., Kazienko D., *Housing of the detector of diagnostic signals values deviations*. Polish Patent Office, Wp.27118, Registred design nr 25673.
3. Nozdrzykowski, K. *Device for measuring positional deviation of axis of crankshaft pivot set*. Polish Patent Office, PL393829-A1; PL218653-B1.

## Figures and Tables

**Table 1:** Calculated coefficients of the monoharmonic model of reaction forces at main journals of crankshaft S-9285-3600-10-8-149-144-O-01.

Journal	1	2	3	4	5	6	7	8	9	10
*R*_0_ (N)	780.17	881.40	997.47	1032.26	984.30	953.88	985.50	1018.80	1070.50	580.07
*C_R_*_2_ (N)	52.69	124.04	174.53	220.96	188.99	169.82	188.28	212.82	136.54	34.52
*ϕ_R_*_2_ (deg)	246	60	224	37	224	55	224	35	217	42

**Table 2:** Model’s fit and maximum relative errors for main journals of the crankshaft S-9285-3600-10-8-149-144-O-01.

Journal	1	2	3	4	5	6	7	8	9	10
*R*^2^ (-)	1.0000	1.0000	0.9999	1.0000	1.0000	1.0000	1.0000	1.0000	1.0000	1.0000
*δ* (%)	0.0579	0.0735	0.7211	0.2029	0.7788	0.1468	0.1513	0.1513	0.1118	0.1382

**Table 3:** Calculated coefficients of the monoharmonic model of reaction forces at main journals of the crankshaft S-8658-3600-10-8-149-114-O-02.

Journal	1	2	3	4	5	6	7	8	9	10
*R*_0_ (N)	782.93	816.13	935.03	951.08	913.18	870.04	915.33	942.25	975.95	556.39
*C_R_*_2_ (N)	71.1	153.5	200.25	248.9	214.25	191.25	213.65	241.35	159.05	42.9
*ϕ_R_*_2_ (deg)	243	57	223	38	225	53	225	37	217	40

**Table 4:** Model’s fit and maximum relative errors for main journals of the crankshaft S-8658-3600-10-8-149-114-O-02.

Journal	1	2	3	4	5	6	7	8	9	10
*R*^2^ (-)	0.9999	1.0000	1.0000	1.0000	1.0000	1.0000	1.0000	1.0000	1.0000	1.0000
*δ* (%)	0.0759	0.0726	0.7760	0.3426	1.0481	0.2239	1.0351	0.2567	0.1566	0.1253

**Table 5:** Calculated coefficients of the monoharmonic model of reaction forces at main journals of the crankshaft S-16942-3600-10-8-149-144-C-03.

Journal	1	2	3	4	5	6	7	8	9	10
*R*_0_ (N)	636.94	1459.39	1998.68	2058.99	1874.93	1904.53	1875.13	2040.33	2144.96	947.85
*C_R2_* (N)	97.65	238.70	346.95	446.75	383.85	333.40	382.85	433.55	274.05	70.95
*ϕ_R_*_2_ (deg)	247	59	222	35	223	54	223	33	212	34

**Table 6:** Model’s fit and maximum relative errors for main journals of the crankshaft S-16942-3600-10-8-149-144-C-03.

Journal	1	2	3	4	5	6	7	8	9	10
*R*^2^ (-)	1.0000	1.0000	1.0000	0.9999	1.0000	1.0000	1.0000	1.0000	1.0000	1.0000
*δ* (%)	0.1748	0.1053	0.4610	0.1922	0.6581	0.1469	0.6564	0.1089	0.0661	0.0413

**Table 7:** Calculated coefficients of the monoharmonic model of reaction forces at main journals of the crankshaft S-12075-3600-10-8-149-144-C-04.

Journal	1	2	3	4	5	6	7	8	9	10
*R*_0_ (N)	725.58	1110.52	1294.29	1445.64	1307.01	1334.94	1255.87	1435.69	1443.20	722.07
*C_R_*_2_ (N)	68.80	166.36	264.50	330.56	276.49	238.18	283.67	321.66	204.46	53.14
*ϕ_R_*_2_ (deg)	239	51	218	33	221	50	219	31	210	32

**Table 8:** Model’s fit and maximum relative errors for main journals of the crankshaft S-12075-3600-10-8-149-144-C-04.

Journal	1	2	3	4	5	6	7	8	9	10
*R*^2^ (-)	0.9999	1.0000	1.0000	0.9999	1.0000	1.0000	0.9999	1.0000	1.0000	0.9999
*δ* (%)	0.0706	0.2168	0.2763	0.2052	0.5482	0.3299	0.4426	0.0542	0.0487	0.0588

**Table 9:** Calculated coefficients of the monoharmonic model of reaction forces at main journals of the crankshaft S-9283-3600-10-8-149-144-O-05.

Journal	1	2	3	4	5	6	7	8	9	10
*R*_0_ (N)	747.64	918.19	990.79	1034.98	986.30	947.27	987.34	1032.79	1035.96	601.33
*C_R_*_2_ (N)	51.05	113.85	145.53	171.88	133.92	90.08	136.82	168.35	106.89	29.47
*ϕ_R_*_2_ (deg)	248	58	210	1	163	0	198	3	166	332

**Table 10:** Model’s fit and maximum relative errors for main journals of the crankshaft S-9283-3600-10-8-149-144-O-05.

Journal	1	2	3	4	5	6	7	8	9	10
*R*^2^ (-)	1.0000	1.0000	0.9999	1.0000	1.0000	0.9999	1.0000	0.9999	1.0000	1.0000
*δ* (%)	0.0713	0.0086	0.1208	0.0046	0.4026	0.0704	0.3515	0.1262	0.3415	0.0114

**Table 11:** Calculated coefficients of the monoharmonic model of reaction forces at main journals of the crankshaft S-9283-3600-10-8-149-144-O-06.

Journal	1	2	3	4	5	6	7	8	9	10
*R*_0_ (N)	892.40	751.37	1088.55	882.24	1097.81	878.18	1101.47	872.23	1146.03	572.31
*C_R_*_2_ (N)	40.39	90.09	101.45	104.52	103.24	101.03	100.60	91.53	59.37	17.87
*ϕ_R_*_2_ (deg)	269	89	268	89	270	88	267	90	273	93

**Table 12:** Model’s fit and maximum relative errors for main journals of the crankshaft S-9283-3600-10-8-149-144-O-06.

Journal	1	2	3	4	5	6	7	8	9	10
*R*^2^ (-)	1.0000	0.9999	1.0000	1.0000	1.0000	1.0000	1.0000	1.0000	1.0000	1.0000
*δ* (%)	0.0178	0.0887	0.0654	0.0795	0.0246	0.0578	0.0575	0.0189	0.0312	0.0123

**Table 13:** Calculated coefficients of the monoharmonic model of reaction forces for main journals of the crankshaft S-9283-3600-10-8-149-144-O-07.

Journal	1	2	3	4	5	6	7	8	9	10
*R*_0_ (N)	752.76	895.23	1032.15	1024.88	916.93	1025.31	1023.51	904.80	1129.57	577.39
*C_R_*_2_ (N)	47.79	106.74	125.58	96.40	29.89	96.19	95.13	37.92	66.79	30.90
*ϕ_R_*_2_ (deg)	257	73	245	50	146	249	51	155	271	83

**Table 14:** Model’s fit and maximum relative errors for main journals of the crankshaft S-9283-3600-10-8-149-144-O-07.

Journal	1	2	3	4	5	6	7	8	9	10
*R*^2^ (-)	1.0000	0.9999	1.0000	1.0000	0.9999	1.0000	1.0000	1.0000	0.9999	1.0000
*δ* (%)	0.1934	0.3983	0.0982	0.1577	0.0280	0.1276	0.1261	0.0226	0.0478	0.0421

**Table 15:** Calculated coefficients of the monoharmonic model of reaction forces for main journals of the crankshaft S-8479-3600-10-8-149-144-O-08.

Journal	1	2	3	4	5	6	7	8	9	10
*R*_0_ (N)	779.92	789.67	912.92	916.33	895.67	863.50	896.17	904.08	972.00	549.00
*C_R_*_2_ (N)	36.00	80.50	113.00	143.50	120.00	105.50	119.50	137.00	89.00	23.00
*ϕ_R_*_2_ (deg)	246	58	222	37	225	55	225	35	213	35

**Table 16:** Model’s fit and maximum relative errors for main journals of the crankshaft S-8479-3600-10-8-149-144-O-08.

Journal	1	2	3	4	5	6	7	8	9	10
*R*^2^ (-)	0.9999	1.0000	1.0000	1.0000	1.0000	0.9999	0.9999	1.0000	1.0000	0.9999
*δ* (%)	0.1056	0.0821	0.3048	0.1585	0.4840	0.1391	0.4811	0.1087	0.0715	0.0519

**Table 17:** Calculated coefficients of the monoharmonic model of reaction forces for main journals of the crankshaft S-7051-3600-10-8-149-114-Z-09.

Journal	1	2	3	4	5	6	7	8	9	10
*R*_0_ (N)	817.63	670.72	723.53	742.93	715.18	683.13	715.45	736.93	755.70	490.03
*C_R_*_2_ (N)	58.95	125.40	156.90	190.60	164.35	147.35	163.95	183.95	121.20	32.75
*ϕ_R_*_2_ (deg)	242	56	224	39	225	53	225	38	218	41

**Table 18:** Model’s fit and maximum relative errors for main journals of the crankshaft S-7051-3600-10-8-149-114-Z-09.

Journal	1	2	3	4	5	6	7	8	9	10
*R*^2^ (-)	1.0000	0.9999	1.0000	1.0000	1.0000	1.0000	1.0000	1.0000	1.0000	1.0000
*δ* (%)	0.0477	0.1537	0.8284	0.4294	1.0228	0.2191	1.0130	0.3207	0.1859	0.1279

**Table 19:** Calculated coefficients of the monoharmonic model of reaction forces for main journals of the crankshaft S-1977-0740-3-2-149-144-O-10.

Journal	1	2	3
*R*_0_ (N)	388.82	1199.63	388.88
*C_R_*_2_ (N)	19.55	39.10	19.55
*φ_R_*_2_ (deg)	271	91	271

**Table 20:** Model’s fit and maximum relative errors for main journals of the crankshaft S-1977-0740-3-2-149-144-O-10.

Journal	1	2	3
*R*^2^ (-)	1.0000	1.0000	1.0000
*δ* (%)	0.0159	0.0096	0.0149

**Table 21:** Calculated coefficients of the monoharmonic model of reaction forces for main journals of the crankshaft S-1977-0740-3-2-149-144-O-11.

Journal	1	2	3
*R*_0_ (N)	401.47	1173.83	401.48
*C_R_*_2_ (N)	36.27	72.53	36.27
*ϕ_R_*_2_ (deg)	90	270	90

**Table 22:** Model’s fit and maximum relative errors for main journals of the crankshaft S-1977-0740-3-2-149-144-O-11.

Journal	1	2	3
*R*^2^ (-)	0.9997	0.9997	0.9997
*δ* (%)	0.2077	0.1416	0.2077

**Table 23:** Calculated coefficients of the monoharmonic model of reaction forces for main journals of the crankshaft S-1977-0740-3-2-149-144-O-12.

Journal	1	2	3
*R*_0_ (N)	420.35	1136.60	420.40
*C_R2_* (N)	42.76	85.50	42.78
*ϕ_R_*_2_ (degree)	133	313	133

**Table 24:** Model’s fit and maximum relative errors for main journals of the crankshaft S-1977-0740-3-2-149-144-O-12.

Journal	1	2	3
*R*^2^ (-)	1.0000	1.0000	1.0000
*δ* (%)	0.3099	0.2700	0.3143

**Table 25:** The comparison of model fit values and maximum relative errors for the shafts analyzed.

Crankshaft	*R*^2^_min_ (-)	*δ*_max_ (%)
S-9283-3600-10-8-149-144-O-01	0.9999	0.7788
S-8658-3600-10-8-149-114-O-02	0.9999	1.0481
S-16942-3600-10-8-149-144-C-03	0.9999	0.6581
S-12075-3600-10-8-149-144-C-04	0.9999	0.5482
S-9283-3600-10-8-149-144-O-05	0.9999	0.4026
S-9283-3600-10-8-149-144-O-06	0.9999	0.0887
S-9283-3600-10-8-149-144-O-07	0.9999	0.3983
S-8479-3600-10-8-149-144-O-08	0.9999	0.4840
S-7051-3600-10-8-149-144-Z-09	0.9999	1.0228
S-1977-0740-3-2-149-144-O-10	1.0000	0.0159
S-1977-0740-3-2-149-144-O-11	0.9997	0.2077
S-1977-0740-3-2-149-144-O-12	1.0000	0.3143

## Appendix A

% Recursive algorithm
clear,clc,format compact
FiFEA=[0:15:345];%whole turn of the shaft 360 deg, every 15 deg -> reaction forces 0–345 deg
%example shaft: S-12075-3600-10-8-149-144-C-04, example main journal: #10, FEA reaction forces:
RFEA=[749.87 768.80 775.20 767.37 747.40 720.64 694.26 675.33 668.93 676.77 696.74 723.50 749.87 768.80 775.20 767.37 747.40 720.64 694.26 675.33 668.93 676.77 696.74 723.50];

DeltaFi=1;% calculation step (deg)
R0=mean(RFEA);
disp([‘Mean value of the reaction forces: ‘ num2str(R0) ‘ N’])
CR2=0.5*(max(RFEA)-min(RFEA));
disp([‘Reaction forces amplitude of the 2nd harmonic: ‘ num2str(CR2) ‘ N’])
for j=1:360 % calculation loop
FiR2=(j-1)*DeltaFi;
P1=RFEA-(R0+CR2*sind(FiFEA*2+FiR2));
Q(j)=P1*P1’; % sum of squares of deviations
end
k=length(Q);
[Qmin,L]=min(Q);
[Qmax,H]=max(Q);
FiR2a=L-1;
FiR2b=H+1;

disp([‘Reaction forces phase shift of the 2nd harmonic: ‘ num2str(FiR2a) ‘ deg’])

## Appendix B

**Table A1:** Reaction forces based on FEA calculations for the crankshaft S-9285-3600-10-8-149-144-O-01.

Angular Position (˚CA)	Main Journal Number (-)									
	1	2	3	4	5	6	7	8	9	10
	Reaction Forces in Main Journals (N)									
0	731.62	988.50	871.12	1166.33	847.89	1093.01	852.04	1142.22	988.15	603.47
15	727.48	1005.43	823.76	1237.24	796.42	1123.70	799.74	1212.93	944.12	613.51
30	737.46	989.14	822.94	1253.23	795.30	1108.89	797.22	1231.62	933.95	614.59
45	758.88	943.98	868.88	1210.01	844.82	1052.54	845.15	1193.29	960.37	606.42
60	786.00	882.05	949.28	1119.16	931.72	969.75	930.69	1108.20	1016.30	591.19
75	811.57	819.94	1042.60	1005.03	1032.70	882.71	1030.91	999.16	1086.75	572.98
90	828.72	774.30	1123.82	898.19	1120.71	814.74	1118.97	895.38	1152.84	556.67
105	832.87	757.35	1171.18	827.29	1172.17	784.05	1171.26	824.67	1196.87	546.62
120	822.89	773.65	1172.00	811.30	1173.29	798.87	1173.78	805.98	1207.04	545.54
135	801.47	818.82	1126.05	854.53	1123.77	855.22	1125.85	844.31	1180.62	553.71
150	774.33	880.76	1045.65	945.37	1036.88	938.00	1040.31	929.40	1124.69	568.94
165	748.76	942.87	952.34	1059.50	935.90	1025.04	940.09	1038.44	1054.24	587.16
180	731.62	988.50	871.12	1166.33	847.89	1093.01	852.04	1142.22	988.15	603.47
195	727.48	1005.43	823.76	1237.24	796.42	1123.70	799.74	1212.93	944.12	613.51
210	737.46	989.14	822.94	1253.23	795.30	1108.89	797.22	1231.62	933.95	614.59
225	758.88	943.98	868.88	1210.01	844.82	1052.54	845.15	1193.29	960.37	606.42
240	786.00	882.05	949.28	1119.16	931.72	969.75	930.69	1108.20	1016.30	591.19
255	811.57	819.94	1042.60	1005.03	1032.70	882.71	1030.91	999.16	1086.75	572.98
270	828.72	774.30	1123.82	898.19	1120.71	814.74	1118.97	895.38	1152.84	556.67
285	832.87	757.35	1171.19	827.29	1172.17	784.05	1171.26	824.67	1196.87	546.62
300	822.89	773.65	1172.00	811.30	1173.29	798.87	1173.78	805.98	1207.04	545.54
315	801.47	818.82	1126.05	854.53	1123.77	855.22	1125.85	844.31	1180.62	553.71
330	774.33	880.76	1045.65	945.37	1036.88	938.00	1040.31	929.40	1124.69	568.94
345	748.76	942.86	952.34	1059.50	935.90	1025.04	940.09	1038.44	1054.24	587.16
360	731.62	988.50	871.12	1166.33	847.89	1093.01	852.04	1142.22	988.15	603.47

**Table A2:** Reaction forces based on FEA calculations for the crankshaft S-8658-3600-10-8-149-114-O-02.

Angular Position (˚CA)	Main Journal Number (-)									
	1	2	3	4	5	6	7	8	9	10
	Reaction Forces in Main Journals (N)									
0	719.80	944.80	793.60	1105.90	757.00	1024.40	760.30	1088.20	880.10	584.20
15	711.80	969.60	737.80	1184.20	699.30	1061.30	702.50	1165.80	828.80	597.20
30	723.00	953.30	734.80	1200.00	698.90	1046.90	701.70	1183.60	816.90	599.30
45	750.20	900.30	785.40	1149.10	756.00	985.20	758.10	1136.60	847.60	589.90
60	786.10	824.70	876.10	1045.10	855.10	892.60	856.70	1037.60	912.70	571.50
75	821.20	746.80	982.60	916.00	969.80	793.90	971.00	913.00	994.80	549.00
90	846.10	687.50	1076.40	796.30	1069.30	715.70	1070.40	796.30	1071.80	528.60
105	854.00	662.60	1132.30	718.00	1127.00	678.80	1128.20	718.70	1123.10	515.60
120	842.90	678.90	1135.30	702.20	1127.40	693.20	1129.00	700.90	1135.00	513.50
135	815.70	732.00	1084.70	753.10	1070.40	754.90	1072.50	747.90	1104.30	522.90
150	779.80	807.60	993.90	857.00	971.30	847.50	973.90	846.90	1039.20	541.30
165	744.60	885.50	887.40	986.10	856.60	946.10	859.70	971.50	957.10	563.70
180	719.80	944.80	793.60	1105.90	757.00	1024.40	760.30	1088.20	880.10	584.20
195	711.80	969.60	737.80	1184.20	699.30	1061.30	702.50	1165.80	828.80	597.20
210	723.00	953.30	734.80	1200.00	698.90	1046.90	701.70	1183.60	816.90	599.30
225	750.10	900.30	785.40	1149.10	756.00	985.20	758.10	1136.60	847.60	589.90
240	786.10	824.70	876.10	1045.10	855.10	892.60	856.70	1037.60	912.70	571.50
255	821.20	746.80	982.60	916.00	969.80	793.90	971.00	913.00	994.80	549.00
270	846.10	687.50	1076.40	796.30	1069.30	715.70	1070.40	796.30	1071.80	528.60
285	854.00	662.60	1132.30	718.00	1127.00	678.80	1128.20	718.70	1123.10	515.60
300	842.90	678.90	1135.30	702.20	1127.40	693.20	1129.00	700.90	1135.00	513.50
315	815.70	732.00	1084.70	753.10	1070.40	754.90	1072.50	747.90	1104.30	522.90
330	779.80	807.60	993.90	857.00	971.30	847.50	973.90	846.90	1039.20	541.30
345	744.60	885.50	887.40	986.10	856.60	946.10	859.70	971.50	957.10	563.70
360	719.80	944.80	793.60	1105.90	757.00	1024.40	760.30	1088.20	880.10	584.20

**Table A3:** Reaction forces based on FEA calculations for the crankshaft S-16942-3600-10-8-149-144-C-03.

Angular Position (˚CA)	Main Journal Number (-)									
	1	2	3	4	5	6	7	8	9	10
	Reaction Forces in Main Journals (N)									
0	546.10	1663.20	1762.90	2313.40	1607.30	2176.70	1608.20	2275.10	2000.90	987.90
15	539.30	1698.10	1662.20	2463.80	1498.80	2237.90	1500.00	2426.20	1903.50	1011.90
30	558.60	1669.00	1651.70	2505.70	1491.10	2209.80	1492.30	2473.90	1870.90	1018.80
45	598.90	1583.70	1734.20	2428.00	1586.20	2099.90	1587.10	2405.40	1911.70	1006.60
60	649.40	1465.10	1887.50	2251.40	1758.60	1937.60	1759.20	2239.10	2015.00	978.70
75	696.60	1345.00	2070.70	2023.20	1962.30	1766.50	1962.30	2019.60	2153.10	942.60
90	727.80	1255.50	2234.50	1804.60	2142.50	1632.40	2142.00	1805.60	2289.10	907.80
105	734.60	1220.70	2335.20	1654.20	2251.00	1571.10	2250.30	1654.50	2386.40	883.80
120	715.30	1249.80	2345.60	1612.20	2258.80	1599.20	2258.00	1606.80	2419.00	876.90
135	675.00	1335.10	2263.20	1690.00	2163.70	1709.20	2163.10	1675.20	2378.20	889.10
150	624.40	1453.70	2109.80	1866.60	1991.20	1871.40	1991.10	1841.50	2274.90	917.00
165	577.30	1573.80	1926.70	2094.80	1787.60	2042.60	1788.00	2061.10	2136.80	953.10
180	546.10	1663.20	1762.90	2313.40	1607.30	2176.70	1608.20	2275.10	2000.90	987.90
195	539.30	1698.10	1662.20	2463.80	1498.80	2237.90	1500.00	2426.20	1903.50	1011.90
210	558.60	1669.00	1651.70	2505.70	1491.10	2209.80	1492.30	2473.90	1870.90	1018.80
225	598.90	1583.70	1734.20	2428.00	1586.20	2099.90	1587.10	2405.40	1911.70	1006.60
240	649.40	1465.10	1887.50	2251.40	1758.60	1937.60	1759.20	2239.10	2015.00	978.70
255	696.60	1345.00	2070.70	2023.20	1962.30	1766.50	1962.30	2019.60	2153.10	942.60
270	727.80	1255.50	2234.50	1804.60	2142.50	1632.40	2142.00	1805.60	2289.10	907.80
285	734.60	1220.70	2335.20	1654.20	2251.00	1571.10	2250.30	1654.50	2386.40	883.80
300	715.30	1249.80	2345.60	1612.20	2258.80	1599.20	2258.00	1606.80	2419.00	876.90
315	675.00	1335.10	2263.20	1690.00	2163.70	1709.20	2163.10	1675.20	2378.20	889.10
330	624.40	1453.70	2109.80	1866.60	1991.20	1871.40	1991.10	1841.50	2274.90	917.00
345	577.30	1573.80	1926.70	2094.80	1787.60	2042.60	1788.00	2061.10	2136.80	953.10
360	546.10	1663.20	1762.90	2313.40	1607.30	2176.70	1608.20	2275.10	2000.90	987.90

**Table A4:** Reaction forces based on FEA calculations for the crankshaft S-12075-3600-10-8-149-144-C-04.

Angular Position (˚CA)	Main Journal Number (-)									
	1	2	3	4	5	6	7	8	9	10
	Reaction Forces in Main Journals (N)									
0	666.85	1241.62	1131.48	1628.25	1120.83	1520.32	1073.28	1600.71	1341.58	749.87
15	656.78	1276.88	1047.58	1741.92	1039.89	1573.12	986.67	1716.67	1266.49	768.80
30	665.14	1267.57	1029.79	1776.19	1030.53	1562.10	972.19	1757.34	1238.75	775.20
45	689.71	1216.17	1082.87	1721.90	1095.24	1490.21	1033.73	1711.83	1265.79	767.37
60	723.88	1136.46	1192.60	1593.57	1216.71	1376.72	1154.78	1592.32	1340.37	747.40
75	758.51	1049.80	1329.59	1425.61	1362.37	1252.03	1302.93	1430.84	1442.50	720.64
90	784.31	979.42	1457.11	1263.01	1493.19	1149.56	1438.46	1270.66	1544.83	694.26
105	794.38	944.17	1541.00	1149.35	1574.13	1096.76	1525.07	1154.70	1619.92	675.33
120	786.02	953.49	1558.78	1115.08	1583.50	1107.78	1539.54	1114.03	1647.66	668.93
135	761.47	1004.88	1505.69	1169.38	1518.78	1179.67	1478.01	1159.54	1620.61	676.77
150	727.30	1084.58	1395.96	1297.70	1397.32	1293.16	1356.95	1279.05	1546.03	696.74
165	692.67	1171.23	1258.99	1465.66	1251.66	1417.85	1208.81	1440.53	1443.90	723.50
180	666.85	1241.62	1131.48	1628.25	1120.83	1520.32	1073.28	1600.71	1341.58	749.87
195	656.78	1276.88	1047.58	1741.92	1039.89	1573.12	986.67	1716.67	1266.49	768.80
210	665.14	1267.57	1029.79	1776.19	1030.53	1562.10	972.19	1757.34	1238.75	775.20
225	689.71	1216.17	1082.87	1721.90	1095.24	1490.21	1033.73	1711.83	1265.79	767.37
240	723.88	1136.46	1192.60	1593.57	1216.71	1376.72	1154.78	1592.32	1340.37	747.40
255	758.51	1049.80	1329.59	1425.61	1362.37	1252.03	1302.93	1430.84	1442.50	720.64
270	784.31	979.42	1457.11	1263.01	1493.19	1149.56	1438.46	1270.66	1544.83	694.26
285	794.38	944.17	1541.00	1149.35	1574.13	1096.76	1525.07	1154.70	1619.92	675.33
300	786.02	953.49	1558.78	1115.08	1583.50	1107.78	1539.54	1114.03	1647.66	668.93
315	761.47	1004.88	1505.69	1169.38	1518.78	1179.67	1478.01	1159.54	1620.61	676.77
330	727.30	1084.58	1395.96	1297.70	1397.32	1293.16	1356.95	1279.05	1546.03	696.74
345	692.67	1171.23	1258.99	1465.66	1251.66	1417.85	1208.81	1440.53	1443.90	723.50
360	666.85	1241.62	1131.48	1628.25	1120.83	1520.32	1073.28	1600.71	1341.58	749.87

**Table A5:** Reaction forces based on FEA calculations for the crankshaft S-9283-3600-10-8-149-144-O-05.

Angular Position (˚CA)	Main Journal Number (-)									
	1	2	3	4	5	6	7	8	9	10
	Reaction Forces in Main Journals (N)									
0	699.84	1014.79	919.05	1037.94	1026.79	947.93	943.38	1042.84	1062.58	587.43
15	696.59	1032.04	865.35	1123.49	955.73	992.88	882.97	1125.67	1005.57	602.29
30	707.02	1018.78	845.25	1185.31	892.87	1025.61	850.51	1183.61	956.70	616.90
45	728.34	978.57	864.15	1206.86	855.04	1037.35	854.72	1201.14	929.07	627.34
60	754.82	922.18	916.99	1182.35	852.38	1024.95	894.47	1173.56	930.08	630.80
75	779.39	864.72	989.59	1118.35	885.60	991.73	959.09	1108.26	959.46	626.37
90	795.44	821.59	1062.52	1032.02	945.81	946.60	1031.29	1022.74	1009.34	615.23
105	798.69	804.34	1116.22	946.47	1016.86	901.65	1091.71	939.91	1066.35	600.37
120	788.26	817.60	1136.32	884.65	1079.73	868.92	1124.16	881.97	1115.22	585.76
135	766.95	857.81	1117.42	863.10	1117.56	857.18	1119.95	864.44	1142.85	575.33
150	740.46	914.20	1064.60	887.61	1120.20	869.58	1080.20	892.02	1141.80	571.86
165	715.90	971.66	991.98	951.61	1087.00	902.80	1015.58	957.32	1112.46	576.29
180	699.84	1014.79	919.05	1037.94	1026.79	947.93	943.38	1042.84	1062.58	587.43
195	696.59	1032.04	865.35	1123.49	955.73	992.88	882.97	1125.67	1005.57	602.29
210	707.02	1018.78	845.25	1185.31	892.87	1025.61	850.51	1183.61	956.70	616.90
225	728.34	978.57	864.15	1206.86	855.04	1037.35	854.72	1201.14	929.07	627.34
240	754.82	922.18	916.99	1182.35	852.38	1024.95	894.47	1173.56	930.08	630.80
255	779.39	864.72	989.59	1118.35	885.60	991.73	959.09	1108.26	959.46	626.37
270	795.44	821.59	1062.52	1032.02	945.81	946.60	1031.29	1022.74	1009.34	615.23
285	798.69	804.34	1116.22	946.47	1016.86	901.65	1091.71	939.91	1066.35	600.37
300	788.26	817.60	1136.32	884.65	1079.73	868.92	1124.16	881.97	1115.22	585.76
315	766.95	857.81	1117.42	863.10	1117.56	857.18	1119.95	864.44	1142.85	575.33
330	740.46	914.20	1064.59	887.61	1120.22	869.58	1080.21	892.02	1141.84	571.86
345	715.90	971.66	991.98	951.61	1087.00	902.80	1015.58	957.32	1112.46	576.29
360	699.84	1014.79	919.05	1037.94	1026.79	947.93	943.38	1042.84	1062.58	587.43

**Table A6:** Reaction forces based on FEA calculations for the crankshaft S-9283-3600-10-8-149-144-O-06.

Angular Position (˚CA)	Main Journal Number (-)									
	1	2	3	4	5	6	7	8	9	10
	Reaction Forces in Main Journals (N)									
0	852.00	841.46	987.10	986.76	994.57	979.21	1000.87	963.76	1086.66	590.18
15	857.14	830.51	999.28	973.32	1008.27	967.19	1012.02	951.58	1095.99	587.28
30	871.73	798.35	1035.37	935.47	1045.96	931.31	1047.15	918.14	1118.73	580.37
45	891.85	753.61	1085.72	883.36	1097.54	881.20	1096.82	872.40	1148.78	571.31
60	912.12	708.26	1136.82	830.95	1149.20	830.28	1147.74	826.61	1178.09	562.51
75	927.11	674.47	1174.99	792.28	1187.08	792.20	1186.26	793.04	1198.82	556.33
90	932.79	661.28	1190.00	777.71	1201.05	777.15	1202.06	780.70	1205.40	554.44
105	927.65	672.23	1177.83	791.16	1187.35	789.18	1190.91	792.88	1196.07	557.33
120	913.07	704.39	1141.73	829.00	1149.66	825.05	1155.79	826.32	1173.33	564.24
135	892.94	749.13	1091.38	881.11	1098.08	875.16	1106.11	872.07	1143.28	573.31
150	872.67	794.48	1040.28	933.53	1046.42	926.08	1055.19	917.86	1113.97	582.11
165	857.69	828.27	1002.11	972.19	1008.54	964.17	1016.67	951.42	1093.24	588.28
180	852.00	841.46	987.10	986.76	994.57	979.21	1000.87	963.76	1086.66	590.18
195	857.14	830.51	999.28	973.32	1008.27	967.19	1012.02	951.58	1095.99	587.28
210	871.73	798.35	1035.37	935.47	1045.96	931.31	1047.15	918.14	1118.73	580.37
225	891.85	753.61	1085.72	883.36	1097.54	881.20	1096.82	872.40	1148.78	571.31
240	912.12	708.26	1136.82	830.95	1149.20	830.28	1147.74	826.61	1178.09	562.51
255	927.11	674.47	1174.99	792.28	1187.08	792.20	1186.26	793.04	1198.82	556.33
270	932.79	661.28	1190.00	777.71	1201.05	777.15	1202.06	780.70	1205.40	554.44
285	927.65	672.23	1177.83	791.16	1187.35	789.18	1190.91	792.88	1196.07	557.33
300	913.07	704.39	1141.73	829.00	1149.66	825.05	1155.79	826.32	1173.33	564.24
315	892.94	749.13	1091.38	881.11	1098.08	875.16	1106.11	872.07	1143.28	573.31
330	872.67	794.48	1040.28	933.53	1046.42	926.08	1055.19	917.86	1113.97	582.11
345	857.69	828.27	1002.11	972.19	1008.54	964.17	1016.67	951.42	1093.24	588.28
360	852.00	841.46	987.10	986.76	994.57	979.21	1000.87	963.76	1086.66	590.18

**Table A7:** Reaction forces based on FEA calculations for the crankshaft S-9283-3600-10-8-149-144-O-07.

Angular Position (˚CA)	Main Journal Number (-)									
	1	2	3	4	5	6	7	8	9	10
	Reaction Forces in Main Journals (N)									
0	704.97	1000.45	918.33	1100.18	933.89	934.39	1098.46	920.77	1062.78	608.29
15	705.70	1001.97	906.56	1121.28	919.26	929.13	1118.64	901.35	1072.58	606.06
30	719.03	974.88	928.44	1116.55	904.00	949.64	1113.33	882.86	1097.65	596.14
45	741.40	926.46	978.11	1087.25	892.21	990.43	1083.95	870.24	1131.27	581.20
60	766.81	869.66	1042.26	1041.24	887.04	1040.57	1038.38	866.88	1164.44	565.24
75	788.46	819.71	1103.70	990.85	889.88	1086.62	988.83	873.69	1188.26	552.54
90	800.54	790.00	1145.96	949.57	899.96	1116.24	948.57	888.83	1196.35	546.50
105	799.82	788.48	1157.73	928.48	914.59	1121.50	928.39	908.25	1186.55	548.73
120	786.49	815.57	1135.85	933.21	929.85	1100.99	933.70	926.74	1161.48	558.65
135	764.12	864.00	1086.18	962.51	941.64	1060.20	963.07	939.36	1127.86	573.59
150	738.70	920.79	1022.03	1008.51	946.82	1010.06	1008.64	942.71	1094.69	589.55
165	717.05	970.74	960.60	1058.91	943.98	964.01	1058.20	935.91	1070.87	602.25
180	704.97	1000.45	918.33	1100.18	933.89	934.39	1098.46	920.77	1062.78	608.29
195	705.70	1001.97	906.56	1121.28	919.26	929.13	1118.64	901.35	1072.58	606.06
210	719.03	974.88	928.44	1116.55	904.00	949.64	1113.33	882.86	1097.65	596.14
225	741.40	926.46	978.11	1087.25	892.21	990.43	1083.95	870.24	1131.27	581.20
240	766.81	869.66	1042.26	1041.24	887.04	1040.57	1038.38	866.88	1164.44	565.24
255	788.46	819.71	1103.70	990.85	889.88	1086.62	988.83	873.69	1188.26	552.54
270	800.54	790.00	1145.96	949.57	899.96	1116.24	948.57	888.83	1196.35	546.50
285	799.82	788.48	1157.73	928.48	914.59	1121.50	928.39	908.25	1186.55	548.73
300	786.49	815.57	1135.85	933.21	929.85	1100.99	933.70	926.74	1161.48	558.65
315	764.12	864.00	1086.18	962.51	941.64	1060.20	963.07	939.36	1127.86	573.59
330	738.70	920.79	1022.03	1008.51	946.82	1010.06	1008.64	942.71	1094.69	589.55
345	717.05	970.74	960.60	1058.91	943.98	964.01	1058.20	935.91	1070.87	602.25
360	704.97	1000.45	918.33	1100.18	933.89	934.39	1098.46	920.77	1062.78	608.29

**Table A8:** Reaction forces based on FEA calculations for the crankshaft S-8479-3600-10-8-149-144-O-08.

Angular Position (˚CA)	Main Journal Number (-)									
	1	2	3	4	5	6	7	8	9	10
	Reaction Forces in Main Journals (N)									
0	747.00	858.00	835.00	1003.00	808.00	951.00	810.00	983.00	923.00	562.00
15	744.00	870.00	803.00	1049.00	776.00	969.00	777.00	1029.00	893.00	570.00
30	750.00	861.00	800.00	1060.00	776.00	959.00	777.00	1041.00	883.00	572.00
45	765.00	833.00	827.00	1032.00	808.00	924.00	808.00	1017.00	898.00	568.00
60	784.00	793.00	877.00	973.00	864.00	872.00	863.00	962.00	932.00	559.00
75	801.00	752.00	937.00	899.00	928.00	818.00	927.00	892.00	977.00	547.00
90	813.00	721.00	990.00	830.00	984.00	776.00	983.00	825.00	1021.00	536.00
105	816.00	709.00	1023.00	783.00	1016.00	758.00	1015.00	780.00	1051.00	528.00
120	809.00	718.00	1026.00	773.00	1015.00	768.00	1016.00	767.00	1061.00	526.00
135	795.00	747.00	999.00	801.00	983.00	803.00	984.00	791.00	1046.00	530.00
150	776.00	787.00	949.00	859.00	927.00	855.00	929.00	846.00	1012.00	539.00
165	759.00	827.00	889.00	934.00	863.00	909.00	865.00	916.00	967.00	551.00
180	747.00	858.00	835.00	1003.00	808.00	951.00	810.00	983.00	923.00	562.00
195	744.00	870.00	803.00	1049.00	776.00	969.00	777.00	1029.00	893.00	570.00
210	750.00	861.00	800.00	1060.00	776.00	959.00	777.00	1041.00	883.00	572.00
225	765.00	833.00	827.00	1032.00	808.00	924.00	808.00	1017.00	898.00	568.00
240	784.00	793.00	877.00	973.00	864.00	872.00	863.00	962.00	932.00	559.00
255	801.00	752.00	937.00	899.00	928.00	818.00	927.00	892.00	977.00	547.00
270	813.00	721.00	990.00	830.00	984.00	776.00	983.00	825.00	1021.00	536.00
285	816.00	709.00	1023.00	783.00	1016.00	758.00	1015.00	780.00	1051.00	528.00
300	809.00	718.00	1026.00	773.00	1015.00	768.00	1016.00	767.00	1061.00	526.00
315	795.00	747.00	999.00	801.00	983.00	803.00	984.00	791.00	1046.00	530.00
330	776.00	787.00	949.00	859.00	927.00	855.00	929.00	846.00	1012.00	539.00
345	759.00	827.00	889.00	934.00	863.00	909.00	865.00	916.00	967.00	551.00
360	747.00	858.00	835.00	1003.00	808.00	951.00	810.00	983.00	923.00	562.00

**Table A9:** Reaction forces based on FEA calculations for the crankshaft S-7051-3600-10-8-149-144-Z-09.

Angular Position (˚CA)	Main Journal Number (-)									
	1	2	3	4	5	6	7	8	9	10
	Reaction Forces in Main Journals (N)									
0	765.70	775.40	611.20	863.50	594.90	801.60	595.70	850.70	680.70	511.90
15	758.70	796.10	568.10	922.60	550.90	830.50	551.70	908.80	642.40	521.50
30	767.40	783.20	566.60	933.50	550.80	819.90	551.50	920.90	634.50	522.80
45	789.60	740.20	607.20	893.30	594.80	772.70	595.30	883.70	659.10	515.30
60	819.30	678.60	679.00	812.90	671.10	701.40	671.30	807.10	709.60	501.00
75	848.60	614.80	762.70	713.70	759.20	625.30	759.10	711.80	772.40	483.70
90	869.60	566.00	835.90	622.30	835.50	564.70	835.20	623.20	830.70	468.20
105	876.60	545.30	879.00	563.30	879.50	535.80	879.20	565.00	869.00	458.50
120	867.90	558.20	880.40	552.30	879.50	546.30	879.40	553.00	876.90	457.30
135	845.60	601.30	839.80	592.50	835.50	593.60	835.60	590.20	852.30	464.80
150	815.90	662.90	768.10	673.00	759.30	664.80	759.60	666.70	801.80	479.10
165	786.70	726.60	684.40	772.20	671.20	741.00	671.80	762.10	739.00	496.30
180	765.70	775.40	611.20	863.50	594.90	801.60	595.70	850.70	680.70	511.90
195	758.70	796.10	568.10	922.60	550.90	830.50	551.70	908.80	642.40	521.50
210	767.40	783.20	566.60	933.50	550.80	819.90	551.50	920.90	634.50	522.80
225	789.60	740.20	607.20	893.30	594.80	772.70	595.30	883.70	659.10	515.30
240	819.30	678.60	679.00	812.90	671.10	701.40	671.30	807.10	709.60	501.00
255	848.60	614.80	762.70	713.70	759.20	625.30	759.10	711.80	772.40	483.70
270	869.60	566.00	835.90	622.30	835.50	564.70	835.20	623.20	830.70	468.20
285	876.60	545.30	879.00	563.30	879.50	535.80	879.20	565.00	869.00	458.50
300	867.90	558.20	880.40	552.30	879.50	546.30	879.40	553.00	876.90	457.30
315	845.60	601.30	839.80	592.50	835.50	593.60	835.60	590.20	852.30	464.80
330	815.90	662.90	768.10	673.00	759.30	664.80	759.60	666.70	801.80	479.10
345	786.70	726.60	684.40	772.20	671.20	741.00	671.80	762.10	739.00	496.30
360	765.70	775.40	611.20	863.50	594.90	801.60	595.70	850.70	680.70	511.90

**Table A10:** Reaction forces based on FEA calculations for the crankshaft S-1977-0740-3-2-149-144-O-0-10.

Angular Position (˚CA)	Main Journal Number (-)		
	1	2	3
	Reaction Forces in Main Journals (N)		
0	369.30	1238.70	369.30
15	372.00	1233.20	372.10
30	379.30	1218.70	379.40
45	389.10	1199.00	389.20
60	398.90	1179.60	398.90
75	405.90	1165.50	406.00
90	408.40	1160.50	408.40
105	405.60	1166.10	405.70
120	398.30	1180.60	398.40
135	388.50	1200.20	388.60
150	378.80	1219.70	378.80
165	371.70	1233.80	371.80
180	369.30	1238.70	369.30
195	372.00	1233.20	372.10
210	379.30	1218.70	379.40
225	389.10	1199.00	389.20
240	398.90	1179.60	398.90
255	405.90	1165.50	406.00
270	408.40	1160.50	408.40
285	405.60	1166.10	405.70
300	398.30	1180.60	398.40
315	388.50	1200.20	388.60
330	378.80	1219.70	378.80
345	371.70	1233.80	371.80
360	369.30	1238.70	369.30

**Table A11:** Reaction forces based on FEA calculations for the crankshaft S-1977-0740-3-2-149-144-O-11.

Angular Position (˚CA)	Main Journal Number (-)		
	1	2	3
	Reaction Forces in Main Journals (N)		
0	438.42	1099.93	438.43
15	433.11	1110.56	433.12
30	419.10	1138.59	419.10
45	400.64	1175.50	400.65
60	382.89	1211.00	382.90
75	370.35	1236.09	370.35
90	365.89	1245.00	365.90
105	370.35	1236.09	370.35
120	383.14	1210.49	383.15
135	401.00	1174.78	401.00
150	419.46	1137.86	419.47
165	433.33	1110.11	433.34
180	438.42	1099.93	438.43
195	433.11	1110.56	433.12
210	419.10	1138.59	419.10
225	400.64	1175.50	400.65
240	382.89	1211.00	382.90
255	370.35	1236.09	370.35
270	365.89	1245.00	365.90
285	370.35	1236.09	370.35
300	383.14	1210.49	383.15
315	401.00	1174.78	401.00
330	419.46	1137.86	419.47
345	433.33	1110.11	433.34
360	438.42	1099.93	438.43

**Table A12:** Reaction forces based on FEA calculations for the crankshaft S-1977-0740-3-2-149-144-O-12.

Angular Position (˚CA)	Main Journal Number (-)		
	1	2	3
	Reaction Forces in Main Journals (N)		
0	452.34	1072.60	452.39
15	433.01	1111.30	433.06
30	410.28	1156.70	410.33
45	390.25	1196.80	390.30
60	378.30	1221.00	378.30
75	377.60	1222.00	377.60
90	388.40	1201.00	388.40
105	407.69	1161.90	407.74
120	430.41	1116.50	430.46
135	450.44	1076.40	450.49
150	462.41	1052.50	462.46
165	463.10	1051.10	463.15
180	452.34	1072.60	452.39
195	433.01	1111.30	433.06
210	410.28	1156.70	410.33
225	390.25	1196.80	390.30
240	378.29	1220.70	378.34
255	377.59	1222.10	377.64
270	388.35	1200.60	388.40
285	407.69	1161.90	407.74
300	430.41	1116.50	430.46
315	450.44	1076.40	450.49
330	462.40	1052.00	462.50
345	463.10	1051.10	463.15
360	452.34	1072.60	452.39
